# Canonical description of wing kinematics and dynamics for a straight flying insectivorous bat (*Hipposideros pratti*)

**DOI:** 10.1371/journal.pone.0218672

**Published:** 2019-06-25

**Authors:** Susheel Sekhar, Peter Windes, Xiaozhou Fan, Danesh K. Tafti

**Affiliations:** Department of Mechanical Engineering, Virginia Tech, Blacksburg, Virginia, United States of America; Texas A&M University System, UNITED STATES

## Abstract

Bats, with highly articulated wings, are some of the most agile flyers in nature. A novel three-dimensional geometric decomposition framework is developed to reduce the complex kinematics of a bat wing into physical movements used to describe flapping flight: namely flapping, stroke plane deviation and pitching, together with cambering and flexion. The decomposition is combined with aerodynamic simulations to investigate the cumulative effect of each motion on force production, and their primary contribution to the unsteady vortex dynamics. For the nearly straight and level flight of *Hipposideros pratti*, results show that the flapping motion by itself induced a moderate drag and lift. Stroke plane deviation increased lift, and nullified the drag. With the inclusion of the pitching motion into the kinematics, lift production further increased by a factor of more than 2.5, and exhibited a positive net thrust by virtue of the favorable wing orientation during the upstroke. The primary contribution of cambering, which included a maximum chord line displacement of ≈40% standard mean chord, was the stabilization of the leading edge vortex during the downstroke. This increased mean lift by about 35% at the expense of net thrust. Flexion was perhaps the most complex motion with maximum displacements of 75% standard mean chord. This was instrumental in reducing the negative lift during the upstroke by preventing the formation of strong leading edge vortices. The aerodynamic effective angle of attack emerged as a heuristic parameter to describe lift and net thrust production across movements.

## Introduction

Flapping of wings is used by the smallest of insects on the millimeter scale to the largest of birds for generating both lift and thrust. Bats distinguish themselves from insects and birds by utilizing a membranous arm-wing that includes the shoulder joint, elbow, wrist, and fingers with phalanges [1], giving them an unparalleled capability to manipulate wing morphology and influence their flight dynamics. In all flying animals, at the most basic level, wing motion described by flapping combined with pitching is sufficient to describe the underlying aerodynamics that generates lift and thrust. Different flyers fine-tune these basic movements by using different phasing between flapping and pitching, deviations from the stroke plane, wing flexibility, passive and active cambering of the wing, wing twist, and so on, to achieve desired functionalities. While several studies have been conducted on insects [2–5] and birds [6] in identifying and isolating some of these physical movements, less progress has been made toward decomposing bat flight kinematics into simpler constituent movements. This can be chiefly attributed to the inherent complexity of bat wing kinematics, but also because of the lack of detailed wing kinematic data and appropriate aerodynamic analysis tools.

The lift and thrust forces generated by a flapping wing have been attributed to a number of fluid dynamic phenomena. Unlike stationary airfoils, which depend on attached steady flow to generate lift, flapping wings depend mostly on unsteady separated flow to generate the required lift and thrust forces. In these unsteady cases, out of the many mechanisms that have been theorized to generate lift and thrust, the most prominent is the mechanism of delayed stall. Here, the leading edge vortices (LEVs), which are generated during stall, are delayed from detaching from the wing surface, and are stabilized and harvested to create force-generating pressure differentials across the wing surface. The stability of the LEV and various factors that affect its longevity have been the topic of many investigations in the literature [7–15]. The most widely accepted explanation of LEV longevity in flapping flight is the presence of a spiral LEV that exhibits an axial flow (spanwise in wing coordinates) in the vortex core, which transports vorticity out of the core and keeps the LEV stable during a large part of the downstroke. While it is commonly acknowledged that most of the heavy lifting in force generation is done by the LEV that forms on the dorsal surface of the wing during the downstroke, other mechanisms such as rotational circulation [16,17], wake capture [18], and clap-and-fling mechanism [15] are also known to contribute to force generation. Rotational circulation generates forces during pronation and supination of the wing based on the Kramer effect. Similarly, wake capture is the process of harvesting wake vortices to generate forces on the wing surface, while clap-and-fling is the process by which vortices are generated at the beginning of the downstroke during pronation by very small insects.

Wing flexibility has been studied extensively in the literature, and has been shown to have a large impact on force generation in flapping flight. Flexibility often manifests as passive cambering and twisting of the wing by inertial and aerodynamic forces to enhance the interaction of the wing surface with the unsteady flow field. Aono et al. [19] showed both experimentally and numerically that in forward flight, passive deformation introduced a twist from the root to the tip, which enhanced thrust production. Wu et al. [20] conducted various experiments to measure the thrust generated by different wing configurations with passive wing deformation and twist. They actuated wings with a single-DOF rotary flapping motion, and found that a rigid wing generated little useful aerodynamic forces, but passive feathering, twisting, and bending of the membrane wings could provide meaningful thrust forces. They showed that chordwise stiffness needs to be orders of magnitude lower than spanwise stiffness to achieve thrust effectiveness and efficiency, and that spanwise stiffness needs to be optimized for different flapping frequency ranges and wing inertias to be efficient. Curet et al. [21] used an idealized wing model consisting of a cantilevered flat plate with a hinged trailing flap, and showed that passive oscillatory motion of this wing significantly increased lift (and drag) as a result of an attached LEV when the freestream velocity exceeded a certain threshold. Gopalakrishnan and Tafti [22] simulated the fluid-structure interaction of a rectangular membrane wing in flapping motion, and showed that spanwise and chordwise flexibility affected the lift and thrust generation. Specifically, they showed that between spanwise and chordwise flexibility, a higher flexibility in the chord yielded higher lift and thrust. The associated flow analysis demonstrated that wing deformation by aerodynamic forces introduced a streamwise camber, which increased the proximity of the LEV to the dorsal surface during the downstroke, increasing both lift and thrust production. Other independent studies [11,23] have also confirmed that the ‘phase lag’ resulting from a purely passive deformation due to chordwise flexibility, where the trailing edge of the wing falls behind the motion of the leading edge, created an effectively larger projected area for the thrust forces to develop. Also in Ref. [11], spanwise flexible wings producing more thrust were associated with a larger effective angle of attack. In an *in vivo* experiment, Mountcastle et al. [24] artificially stiffened the wings of a bumblebee to restrict flexibility, and tested the vertical aerodynamic force production. Observing no significant difference in wingbeat frequency or stroke amplitude after the stiffening, they found that the more rigid wings had an 8.6% reduction in maximum lift in load-lifting trials.

Young et al. [2] and Walker et al. [3] demonstrated that camber and twist are important for desert locusts to achieve better power economy. Nakata et al. [4,25] used an FEM-based thin shell model to account for anisotropic wing stiffness of a hovering hawkmoth, *Manduca sexta*, and found that wing bending (or flexion) delayed the breakdown of the LEV near the wingtip, thus augmenting aerodynamic forces. Wing twist also increased aerodynamic efficiency by favorably orientating the wing. Most interestingly, they found that the spanwise kinematic variation was key to aerodynamic efficiency. Le et al. [26] studied the beetle wing in forward flight and found a similar result: that wing twist significantly increased lift, and that camber variation enhanced the power economy by reducing power consumption, as well as enhancing thrust. More specifically, Zheng et al. [5] pointed out in their comparative study of a Painted Lady butterfly, *Vanessa cardui*, that it was wing twist, not camber, that was the key to performance. Lucas et al. [27] focused their attention on the bending motion of animal wings and fins, and concluded through a statistical analysis across dramatically different taxa that the location of the axis of bending (flexion ratio) and the extent of bending (maximum flexion angle) cluster tightly around 0.65 and 27°, respectively. More recently, Maeda et al. [6] reconstructed the wing kinematics of a hummingbird in terms of spanwise bending, twist and cambering that characterize the flexibility of the wing, along with three angles that describe the entire wing motion. This method allowed them to extract useful and detailed kinematic information about hummingbirds in flight, such as the change in wing area and the twist of the wing.

Compared to other animals, bats excel at maneuvering flight with their highly articulated skeletal structures and thin membranous wings serving as flexible aerodynamic control surfaces [28–30]. Whereas flapping-wing micro aerial vehicles (MAVs) that mimic other animals and/or insects have been extensively researched and adapted to miniaturized flying devices [31–34], equivalent studies and efforts for bat flight have seen very few practical implementations [35,36]. Recent advances in motion capture technology [37,38] have enabled detailed studies of bat flight. Tian et al. [39] experimentally measured the bat wing kinematics and performed a simple analysis of the wake structure for straight flight, which was the first quantitative experimental study that utilized advances in modern wind tunnel technology and developments in flow visualization techniques for bat flight. Muijres et al. [40] showed that a slow flying bat (*Glossophaga soricina*) uses an attached LEV to generate 40% of the lift, using DPIV-derived data. Hubel et al. [41] used a similar experimental approach to reveal four typical vortical structures across different flight speeds of lesser dog-faced fruit bats (*Cynopterus brachyotis*). They discovered that the proportion of the flight cycle without the presence of a tip vortex increased at high speeds, which contrasted with the continuous vortical structure found in bird flight. Subsequently, Hubel et al. [42] compared the kinematics of two insectivorous bats *(Tadarida brasiliensis* and *Myotis velifer*) and showed that they differ significantly in prey pursuit at slow flight. They inferred that *M*. *velifer* has a better flight efficiency for two reasons: a decreased disruption in lift generation between the body and wing, and a characteristic root vortex with diminished strength. Bender et al. [43] and Fan et al. [44] used a high marker density on the wing surface (up to 200 markers on both wings) of an insectivorous bat, Pratt’s roundleaf bat (*H*. *pratti*), which allowed them to extract a more accurate description of the kinematics. Their motion capturing system consisted of a large three dimensional array of 21 cameras (GoPro HERO3+ Black, 720p at 120fps) arranged along the walls of a rectangular flight tunnel. The high fidelity experimental data from this setup has been used to build the bat model in the present work.

Previous numerical studies of bat flight aerodynamics are few, although attempts to apply computational techniques have increased recently. Pivkin et al. [45] made an early attempt to simulate bat flight numerically using a spectral flow solver along with the arbitrary Lagrangian-Eulerian (ALE) method. In their effort, a flying *Pteropus poliocephalus* was simulated at Re = 100, however minimal quantitative analysis was provided from the simulation results. In 2014, Viswanath et al. [46,47] simulated the straight, climbing flight of a fruit bat (*C*. *brachyotis*) using the immersed boundary method. The kinematics of a single wing with 50 markers—borrowed from Riskin et al. [1]—was transformed into a periodic motion to ensure fully developed flow at two different Reynolds numbers (433 and 5625). Visualization of the flow structures provided detailed insight into the effect of the LEV on the generated lift and thrust. Nondimensional lift and drag forces were found to be independent of the Reynolds number in these simulations. Additionally, they showed that by decomposing the kinematics using proper orthogonal decomposition (POD) into the sum of a collection of modes, merely two modes could explain almost all of the resulting averaged forces. Though their decomposition of wing kinematics was based on a single wing, the overall strategy showed excellent potential to identify important kinematics in designing flapping wing MAVs based on bat flight data. In 2015, Wang et al. [48,49] used the immersed boundary method to simulate a slow flying bat at an intermediate Reynolds number (Re = 1000). The wing kinematic data for their simulations—borrowed from Wolf et al. [50]—consisted of only five markers points per wing. It is unclear if five points per wing is sufficient to capture the articulated wing structure and membrane deformation during flight. Additionally, the wing morphology of the model was derived from a *Pteropus poliocephalus* (wingspan ≈ 25 cm, mass ≈ 11 g, ref. [51]), while the kinematics were derived from a *Glossophaga soricina* (wingspan ≈ 117 cm, mass ≈ 770 g, ref. [51]), raising questions about the fidelity of the model. In 2018, Windes et al. conducted a numerical investigation of both aerodynamic power and forces of a straight flying *H*. *pratti* using the same kinematic data set as the present work. Time variation of surface area, aerodynamic loads, and aerodynamic power were analyzed in conjunction with vortex dynamics and wing surface pressure coefficient.

The objective of this paper is to decompose bat wing kinematics into the fundamental canonical descriptors of flapping flight, and investigate each movement’s contribution to the cumulative force production during bat flight. The measured wing motion of an insectivorous *H*. *pratti* in nominally straight and level flight is used. The native wing motion is decomposed into a set of canonical descriptors such as stroke plane angle, flapping amplitude, stroke plane deviation angle, pitch angle, together with chordwise camber and wing flexion. With these synthesized kinematics, aerodynamic simulations of the modeled wing in flight are conducted, and details of how each component of wing motion dictates the underlying unsteady vortex dynamics through the manipulation of the LEV and effective aerodynamic angle of attack are investigated.

The paper is organized as follows: the decomposition paradigm is detailed in the Kinematics Decomposition and Analysis section. Each motion of flapping flight is expounded in terms of parameters that define it, and their incorporation into the kinematics is described in a model equation. This is done with an increasing level of complexity. Next, the Aerodynamic Analyses section introduces the governing equations, mesh set-up and the boundary conditions used in the simulations. The effective angle of attack is revisited for this study because it was identified as a key factor in understanding the aerodynamics. This is followed by simulation results, where the effect of the synthesized wing kinematics on the unsteady flow field generated, and ultimately on force production, is investigated. Finally, conclusions are summarized in the Summary and Conclusions section.

## Kinematics decomposition and analysis

Raw wing kinematic data was obtained from a prior study [43,52], in which a 3D optical motion capture system was used to record an adult male *H*. *pratti* in straight flight. The experimental setup consisted of an array of 21 cameras inside a flight tunnel with a cross section of 1.2 m × 1.2 m, and an approximate length of 4 m. The Svoboda multi-camera self-calibration open source MATLAB package was used for the calibration of the camera array [53]. Additional details related to the experimental facility, motion capture system and the kinematic dataset pre-processing set-up are described in the authors prior work [43,52].

**Fig 1:**
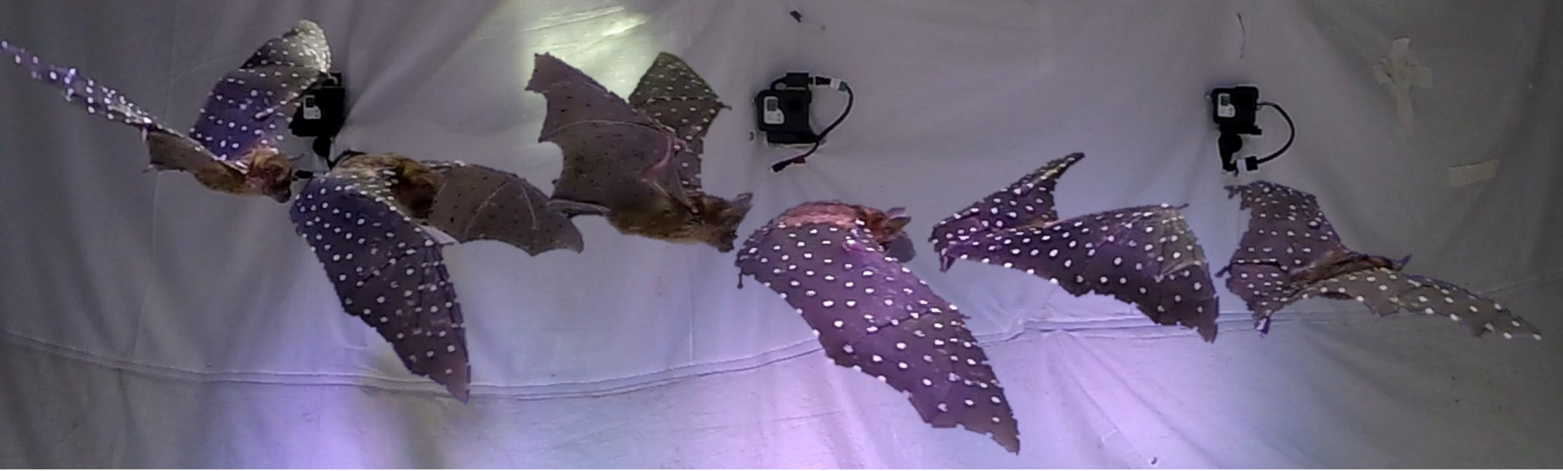
Still-frames showing flight path of *H*. *pratti* in tunnel: Wing marker points recorded and reconstructed to derive wing kinematics.

A 1 m long flight path of a *H*. *pratti* that weighed 55 g, with a wingspan of 52 cm and a standard mean chord of 7.4 cm, over a duration of 0.42 s, was chosen for analysis. During the nearly three flapping cycles recorded, the bat moved laterally by about 5 mm, and descended by about 10 mm at an inclination of approximately 5°. In the absence of any active maneuvers, the recorded flight is considered to be nominally straight and level. Fig 1 shows selected still-frames along the flight path.

### Decomposition of bat wing kinematics using canonical descriptors

The wing kinematic data was decomposed into five types of motion—flapping, stroke plane deviation, pitching, camber, and flexion—which are described in more detail in later sections.

#### Wing kinematics terminology

**Fig 2:**
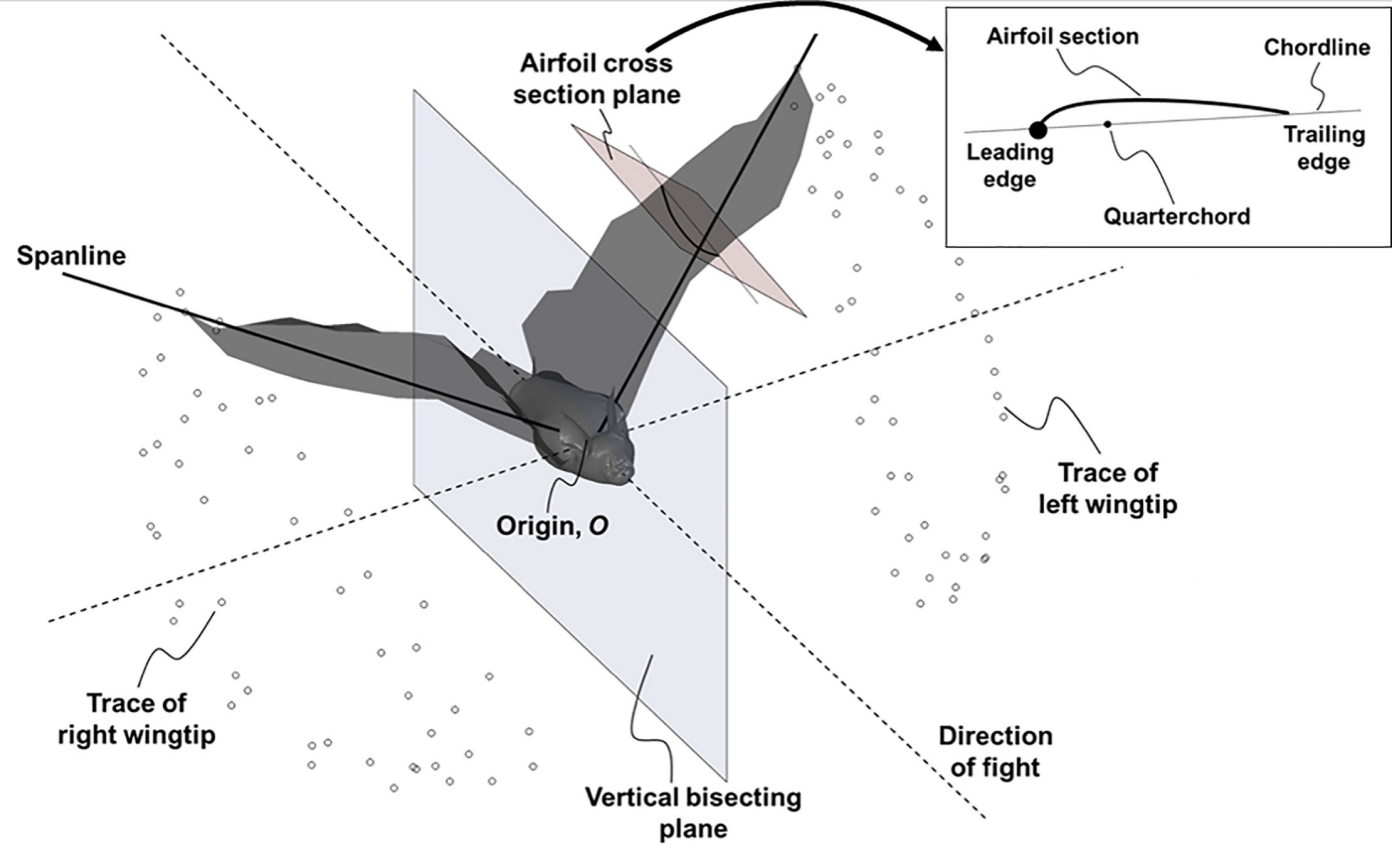
Terminology used in the kinematic decomposition: The right and left wingtips each trace out a locus of points that are projected onto the vertical bisecting plane to determine the stroke plane angle.

The approximate midpoint between the bat’s shoulders is defined as the origin, *O*, which translates through space along with the bat (see Fig 2). The two lines connecting *O* and each of the wingtips are referred to as the *span lines*. The right and left wings each have independent span lines that pivot about the shoulder as the bat flaps and flies.

A cross section of the wing in a plane perpendicular to the span line is referred to as an *airfoil section*, as shown in the inset in Fig 2. Each wing is discretized into a series of airfoils along the span. A straight line connecting the leading and trailing edge of each airfoil is referred to as the airfoil’s *chord line*.

#### Coordinate systems used in kinematic decomposition

**Fig 3:**
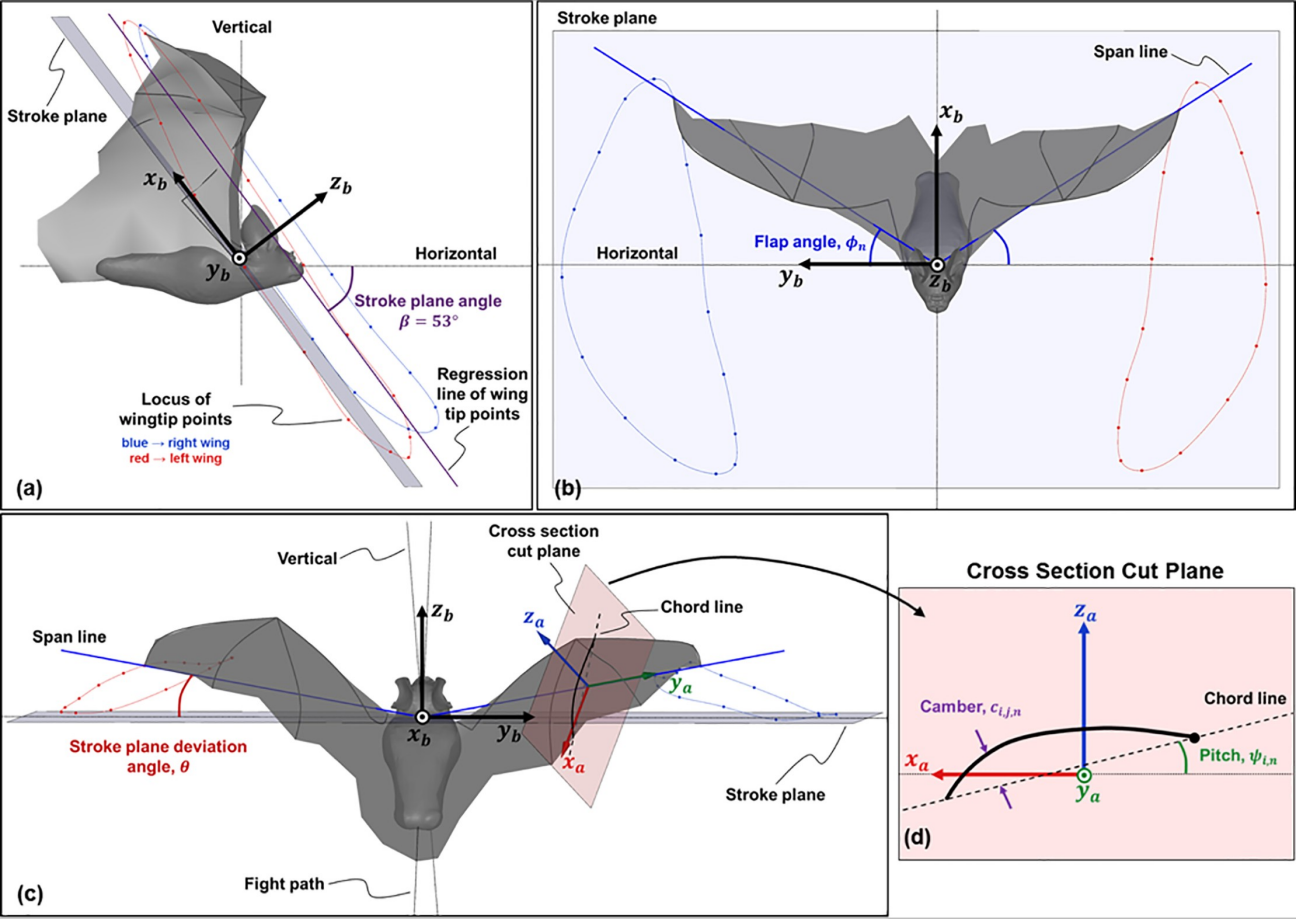
(a) Side view of the bat (view along the y_b_ axis) showing the stroke plane and the body-fixed stroke plane coordinate system (x_b_, y_b_, z_b_), (b) view along the z_b_ axis, looking directly at the stroke plane: the flap angle, *ϕ*_n_, controls the location of the span line and varies with time, (c) view looking down the stroke plane, along the x_b_ axis: the stroke plane deviation angle, *θ*_n_, defines the position of the span line out of the stroke plane; a representative airfoil coordinate system, (x_a_, y_a_, z_a_), is shown on the right wing, and highlighted in (d). The pitch angle, *ψ*_i,n_, defines the rotation of the airfoil section about the span line, and varies along the span, ‘i’, as well as varies in time, ‘n’. The camber, C_i,j,n_, defines the displacement of the airfoil away from the span line, and varies along the span, ‘i’, chord, ‘j’, and in time, ‘n’.

In order to clearly define the five components of motion, two coordinate systems are used. The first coordinate system, (**x**_**b**_, **y**_**b**_, **z**_**b**_), centered at *O*, is a body-fixed system that is defined relative to the stroke plane. Since the bat wings do not flap in a perfectly planar motion, the stroke plane defines the central tendency of the stroke. It is calculated by first projecting the locus of points traced by the two wingtips onto a vertical plane bisecting the bat’s body, as shown in Fig 2. Then, a linear regression is performed on the projected points in order to obtain the slope of the stroke plane, *β*. The **x**_**b**_ and **y**_**b**_ axes of this body-fixed coordinate system lie in the stroke plane, as shown in Fig 3. The **y**_**b**_ axis is always horizontal, while the **x**_**b**_ axis points behind the bat. The **z**_**b**_ axis is perpendicular to both, and follows the right hand rule. This conforms to the convention used in Ref. [54].

A second frame of reference, (**x**_**a**_, **y**_**a**_, **z**_**a**_), which follows each given airfoil through movements about (**x**_**b**_, **y**_**b**_, **z**_**b**_), is also defined. Since both wings are discretized into multiple airfoils, there are as many (**x**_**a**_, **y**_**a**_, **z**_**a**_) systems as there are airfoil sections. The origin of this airfoil coordinate system is located on the span line, with the **y**_**a**_ axis remaining coincident with the span line as the wings flap. When the span line is horizontal at a zero flap angle, **x**_**a**_ is horizontal and **z**_**a**_ is vertical. As the wings flap, each airfoil coordinate system, (**x**_**a**_, **y**_**a**_, **z**_**a**_), shifts and re-orients based on the rotation of the span line as shown in Fig 3.

#### Five wing motion components

**Fig 4:**
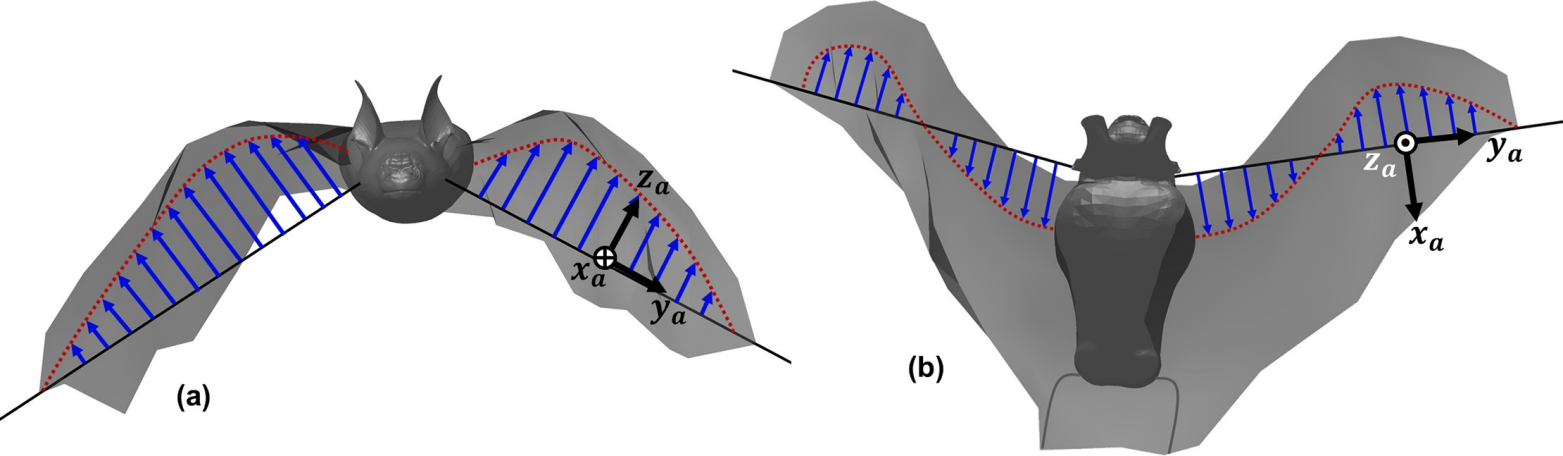
Wing flexion as defined as displacement of the quarterchord from the span line: (a) along the **z**_**a**_ axis, (b) along the **x**_**a**_ axis (refer Fig 3(d) for orientation information).

First, the position of the wing span line is specified using two angles defined relative to the stroke plane. The first angle, *ϕ*, is the flapping angle in the stroke plane (Fig 3(B)), while the second angle, *θ*, captures the deviation of the wing from the stroke plane (Fig 3(C)). A third angle, *ψ*, defined in the airfoil reference frame, captures the rotation of each airfoil about the span line (Fig 3(D)). *ψ* represents the pitch angle of the wing, and can vary both temporally and spatially along the span. The curvature (or camber) of the wing is defined as the displacement of the airfoil from the chord line (Fig 3(D)). The camber can vary temporally, as well as spatially along the span and along the chord. Lastly, flexion of the wing captures the shift of each airfoil section relative to the span line, as shown in Fig 4. Flexion is necessary to properly define wing motion because the quarter-chord of each airfoil does not necessarily remain on the span line. Displacement of the quarter-chord from the span line in the **z**_**a**_ direction is shown in Fig 4(A), while displacement in the **x**_**a**_ direction is shown in Fig 4(B). The overall outline of the decomposition of native kinematics into the five wing motion components is presented as a step-by-step procedure in the Supporting information section (see S1 Text).

Mathematically, the combination of all the aforementioned components is modeled as:

$$W(t=t_{n}){=W}_{i,j,n}=R_{n}(\phi )R_{n}(\theta )\begin{array}{c} R_{i,n}(\psi )[P_{i,j}+C_{i,j,n}+D_{i,n}] \end{array}$$ (1)

Here, subscript ‘n’ signifies a discrete time instant, ‘i’ represents the index that identifies a chord line along the span, and ‘j’ denotes the discretization along each chord line to characterize camber. Thus, **W**(*t* = *t*_n_) represents the combination of every modeled degree of freedom: **R**(*ϕ*) represents the flapping motion of the wing, **R**(*θ*) models stroke plane deviation, and **R**(*ψ*) denotes the pitching motion. It is evident from the equation that **R**(*ϕ*) and **R**(*θ*) vary with time, and are constants for each wing. **R**(*ψ*) is also time-dependent, in addition to varying along the span. **P**_i,j_ = [0,*y*_i_,*z*_i,j_]^T^ corresponds to the base state of the wings consisting of a collection of chord lines. To represent non-zero chordwise cambering, these chord lines transform into airfoil sections with the addition of **C**_i,j,n_ = [*x*_i,j,n_,0,0]^T^. Additionally, **D**_i,n_ = [*x*_i,n_,*y*_i,n_,*z*_i,n_]^T^ denotes wing flexion that represents the displacement of the quarter-chord of each airfoil away from the span line within the airfoil plane (*x*_i,n_,*z*_i,n_), and the spanwise inward-outward movement of the wing (*y*_i,n_).

The following sub-sections use the wing kinematic dataset for a 1 m long straight level flight of *H*. *pratti* to describe the progression from the simplest modeled motion to the most complex combination of movements in detail. For this dataset, the stroke plane angle was estimated to be *β* = 53° over the three flapping cycles.

#### Flapping motion

This motion constitutes the most fundamental of wing movements, flapping. Here, the wings do not deviate from the stroke plane, and the spanwise airfoils do not pitch, nor do they have any camber. Further, the wings do not flex, and thereby, the flapping motion only models the most significant rotational motion of the wing. The governing equation for this simplifies to:

$$W(t=t_{n}){=W}_{i,j,n}=R_{n}(\phi )R_{i,n=0}(\psi )\begin{array}{c} P_{i,j} \end{array}$$ (2)

**Fig 5:**
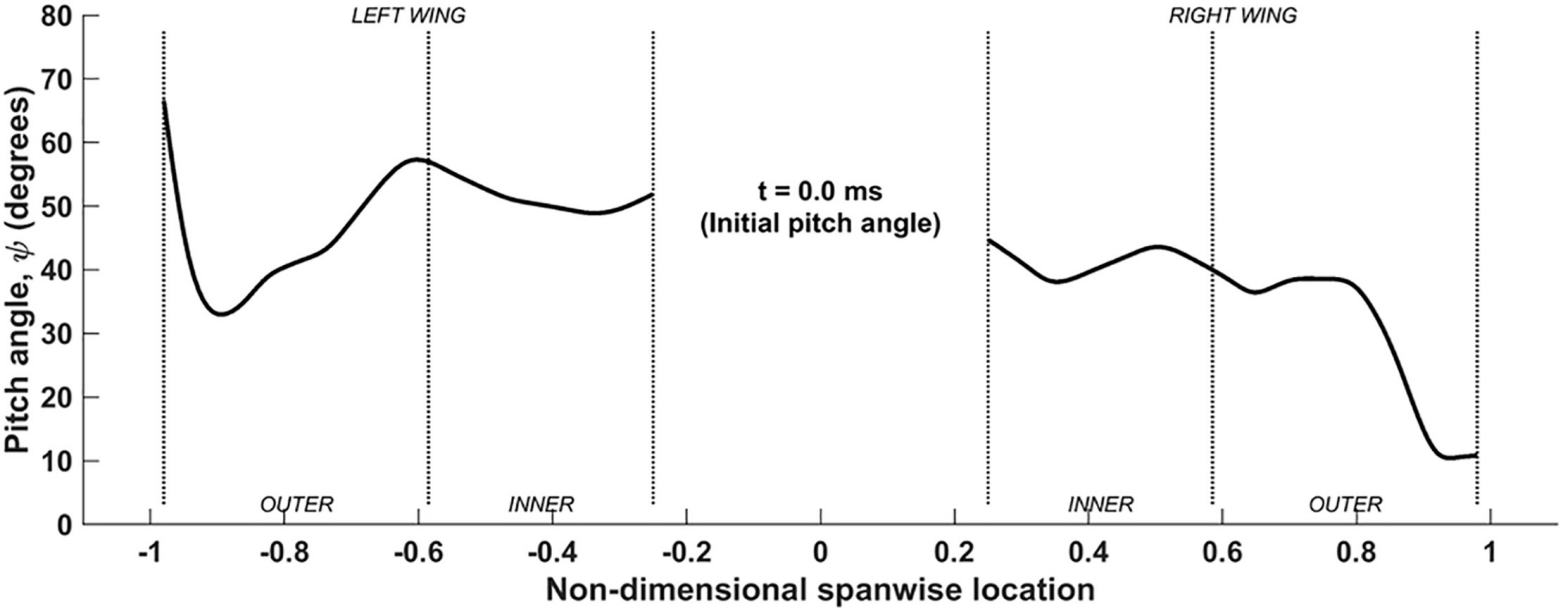
Spanwise variation of pitch angle (*ψ*) at base reference state (time-invariant).

Here, **P**_i,j_ is the time-invariant matrix of points that represents all the chord lines, and **R**_n_(*ϕ*) is the only time-evolving term. **R**_i,n = 0_(*ψ*) is the time-invariant pitching angle distribution along the span used to represent the base state of the wings (Fig 5). Nearly 80% of the left and right wings reveal pitching angles between 40° and 60° and 40° and 50°, respectively. With *β* = 53°, this sets up a nearly horizontal reference state for the wings. There are significant variations at the wingtips, with both wings exhibiting opposite trends in *ψ*. This combination results in the flapping of a rigid, but non-flat wing surface. At each time instant, both wings perform rigid body rotations, and the trajectory of the wingtips form arcs in the stroke plane.

**Fig 6:**
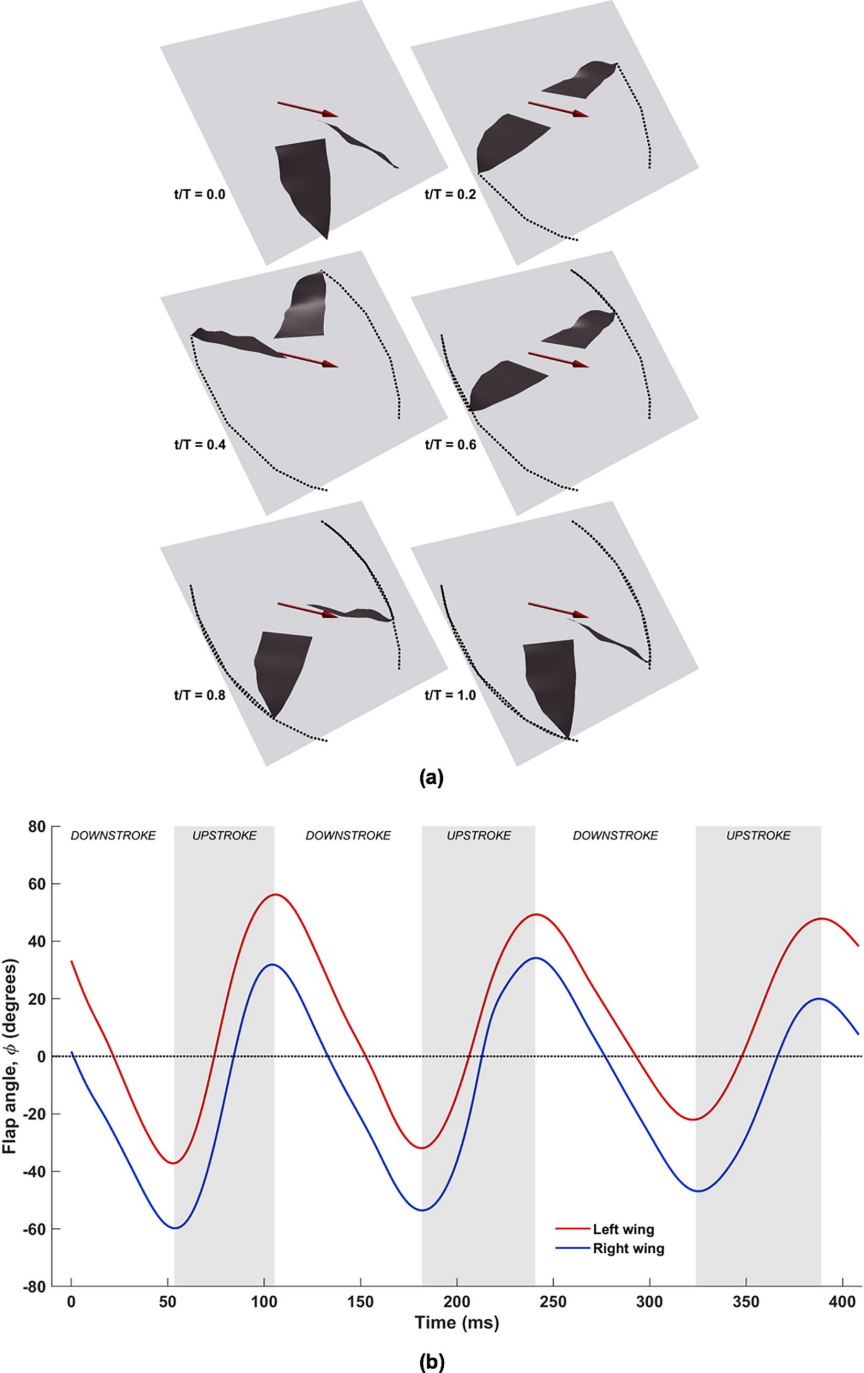
(a) Flapping motion plotted for one flapping cycle (53ms to 182ms), with light-grey tiles representing the stroke plane (x_b_, y_b_) inclined at 53°, dark-grey surfaces representing the wings, tip trajectories traced by dots, and maroon arrows denoting direction of flight; (b) temporal evolution of flapping angle (*ϕ*), with shaded areas representing upstroke.

In Fig 6(A), *t*/T denotes the nondimensional timestamp for one flapping cycle, and the corresponding dimensional time of flight starts at 53 ms and ends at around 182ms in the recorded kinematics. In Fig 6(B), the plot shows the temporal variations of *ϕ* about **y**_**b**_, with a mean amplitude of ≈90°. This is comparable to the flapping amplitude of *C*. *brachyotis* (77°), as noted by Viswanth et al. in their study of straight-climbing flight of fruit bats, and within the range expected of bat species of comparable weight (60°-150° for bats weighing between 0.01-1kg; the *H*. *pratti* in the current study weighed 0.055kg) [47,52]. Whereas the flapping phases of both wings are in perfect synchrony, the angles are different between the right and left wings, with the overall amplitude remaining nearly the same for both. The instantaneous differences in *ϕ* between the left and right wings are attributed to the slight banking towards the left [52]. The overall shape of the temporal evolution of the flapping angle is sinusoidal, and the slope is steeper during the upstroke, which indicates a faster recovery movement.

#### Stroke plane deviation

The stroke plane deviation angle quantifies the extent of in-to and out-of stroke plane motion during the flapping cycles. The governing equation for the wing with this motion included is:

$$W(t=t_{n}){=W}_{i,j,n}={R_{n}(\phi )R}_{n}(\theta )R_{i,n=0}(\psi )P_{i,j}$$ (3)

Here again, **P**_i,j_ is time invariant and represents the chord lines, and **R**_n_(*θ*) is the only time-evolving term. Since flapping constitutes a fundamental motion of the wings, **R**_n_(*ϕ*) is included by default in this, and in subsequent canonical descriptors. The time-invariant reference frame pitching distribution, **R**_i,n = 0_(*ψ*), is also included in this combination.

**Fig 7:**
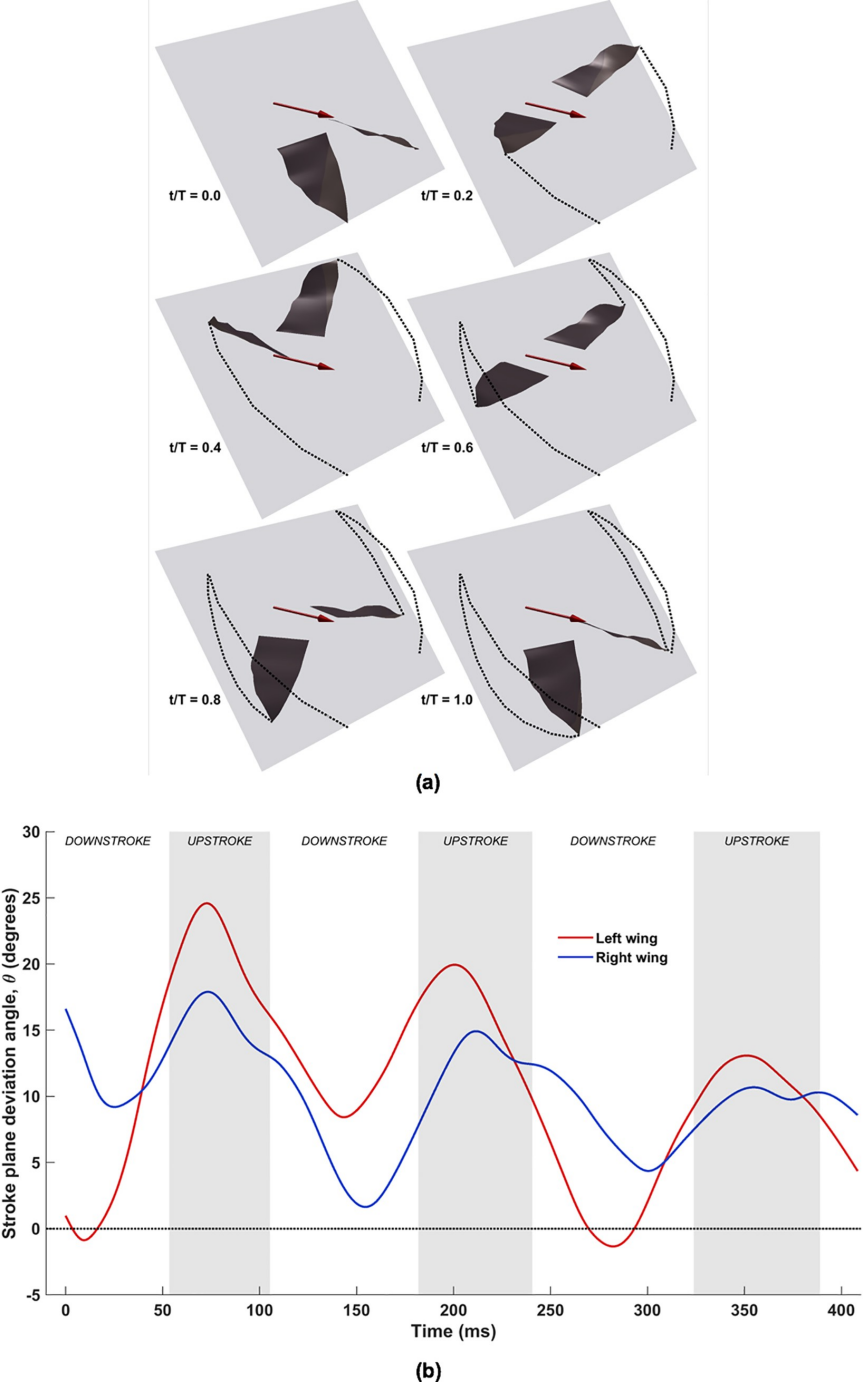
(a) Combined flapping and stroke plane deviation plotted for one flapping cycle (53ms to 182ms), with light-grey tiles representing the stroke plane (**x**_**b**_, **y**_**b**_) inclined at 53°, dark-grey surfaces representing the wings, tip trajectories traced by dots, and maroon arrows denoting direction of flight; (b) temporal evolution of stroke plane deviation angle (*θ***), with shaded areas representing upstroke**.

This combination features each wing performing an additional rigid body rotation out of the stroke plane (**x**_**b**_, **y**_**b**_). Fig 7(A) shows the combined flapping and stroke plane deviation for the same cycle shown for flapping motion. In this case, the wingtips do not lie in the stroke plane, and continually move in and out of the (**x**_**b**_, **y**_**b**_) plane. The combined effect of these two movements (flapping and stroke plane deviation) results in crescent-shaped trajectories. The upstroke is characterized by a backward sweep of the wingtips, followed by a forward sweep during the downstroke to complete the cycle. In Fig 7(B), substantial asymmetry exists between the left and right wings over the three flapping cycles. Peaks and troughs in *θ* occur during the middle of the upstroke and downstroke, respectively, with the left wing lagging behind the right wing. Over the three flapping cycles, the mean peak-to-trough amplitude for the left wing is approximately 13° about the stroke plane, and approximately 9° for the right wing, for nominal stroke deviation angles between ≈0° and 25° (positive angles by virtue of the stroke plane being located near the shoulder, and the wingtips lying ahead)_._ It is notable here that in a previous work of a flapping and pitching 3D rigid flat plate with different stroke plane deviation profiles (O-profile and 8-profile) [55], it was found that the O-profile with *θ* between 10° and 20° produced a near steady thrust over the flapping cycle, leading to the conclusion that it was most suitable for steady forward flight.

#### Pitching motion

The governing equation for the wing kinematics when the pitching motion is included is:

$$W(t=t_{n}){=W}_{i,j,n}=R_{n}(\phi )R_{n}(\theta )\begin{array}{c} R_{i,n}(\psi )P_{i,j} \end{array}$$ (4)

Here too, **P**_i,j_ continues to be time-invariant, whereas **R**_i,n_(*ψ*) now varies with time, and along the span of the wing. **R**_n_(*ϕ*) and **R**_n_(*θ*) are carried over from the previous combination of movements.

**Fig 8:**
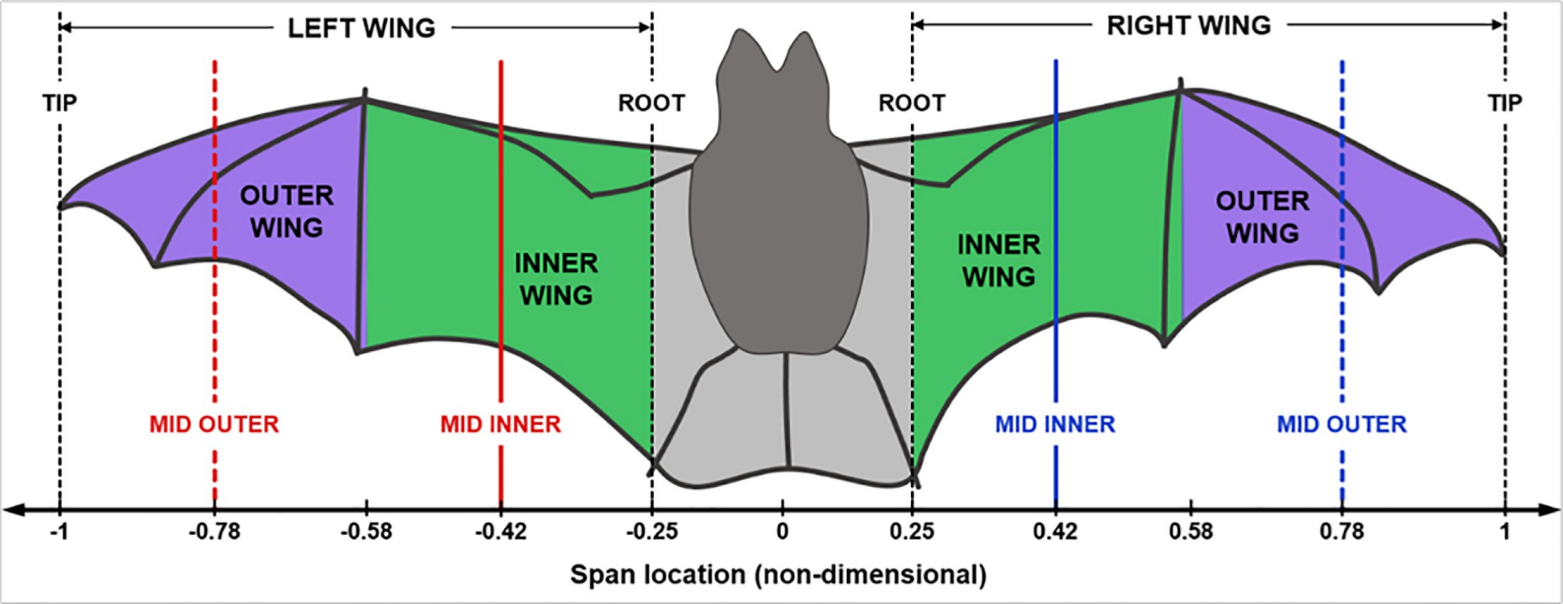
Demarcation of inner (green) and outer (purple) wings based on the instantaneous locations of the left and right wrists, and the location of cutting planes.

The bat wing for this, and subsequent combinations, is assumed to be a flexible membrane that can twist along the span. As a result, different sections along the span exhibit different pitching angles. Fig 8 shows the demarcation of the inner and outer wing sections on both wings that are determined based on the instantaneous locations of the left and right wrists.

**Fig 9:**
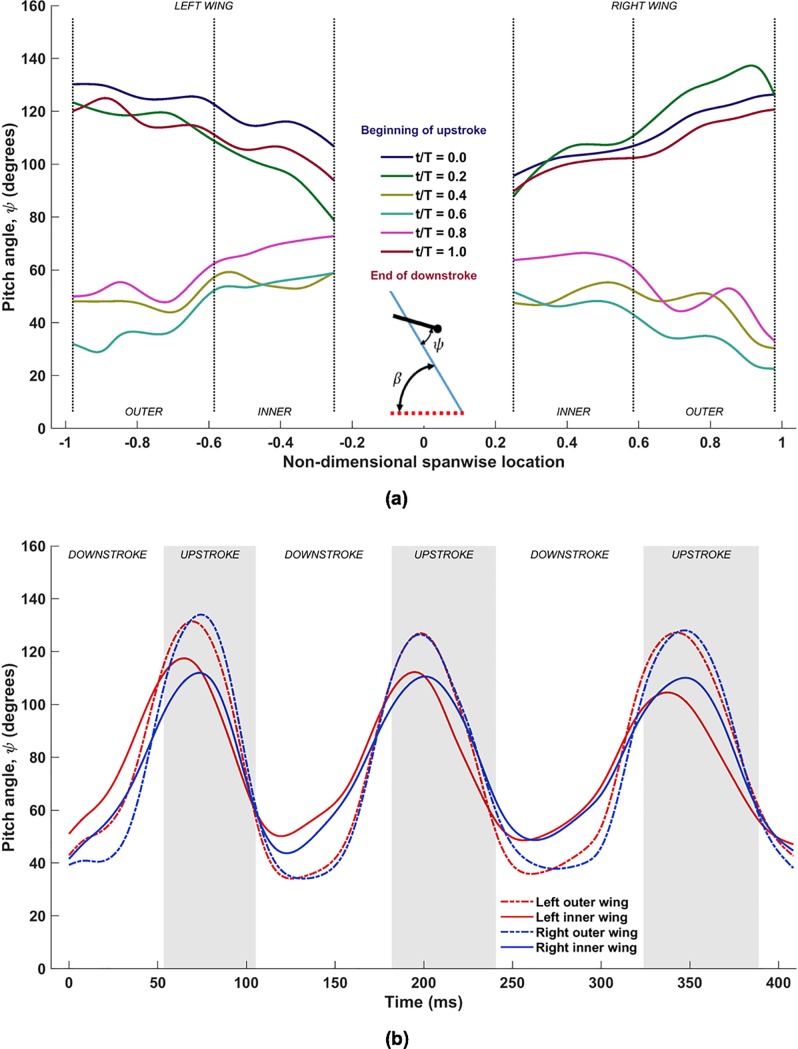
(a) Spanwise variation of pitch angle (*ψ*) for one flapping cycle (53ms to 182ms) with inset showing the definition of pitch angle with respect to stroke plane angle (*β*) for an airfoil chord line; (b) temporal evolution of *ψ* at mid outer and and inner wing sections.

The pitching motion pertains to the orientation of the chord line of each airfoil section with respect to the stroke plane. Fig 9(A) shows the variation of the pitch angle (*ψ*) along the span during one flapping cycle, for the same time-period shown for the previous two movements (53ms to 182ms). Here, the color of the lines denotes the time lapsed from the beginning of the 1^st^ upstroke until the end of the 2^nd^ downstroke, making up one flapping cycle. Fig 9(B) shows the temporal evolution of *ψ* at different locations on the left and right wings identified as the mid-outer and mid-inner lines during the three flapping cycles.

Clearly, *ψ* varies not only with time (Fig 9(B)), but also along the span, which is representative of twist (Fig 9(A)). For much of the flapping cycle, extreme pitch angles are localized towards the ends of the wing (the root and the tip), and a somewhat monotonic and nonlinear variation of the pitching angle is established from the root to the tip, as can be seen in Fig 9(A). The overall variation of *ψ* is also larger in the outer wing (≈100° range) while it is less pronounced in the inner wing (≈60° range). Typically during the upstroke, there is more pronounced twisting of the wing towards higher pitch angles as the bat tends to align the wing with the direction of the upstroke to minimize negative lift [47]. This is apparent from the *t*/T = 0 and 0.2 curves (blue and green lines), which are limited to higher pitch angles. During this time, the pitch angle increases from the root to the tip, maintaining values between 100° to 130°, such that the outer wing is more in alignment with the upward motion of the wings in the stroke plane. Near the end of the upstroke, this trend is reversed: not only do the pitch angles decrease in magnitude across the span of the wing, but now the inner wing exhibits higher pitch angles than the outer wing. From Fig 9(B), during the downstroke, the mid-outer wing reaches a smaller minimum pitch angle (approximately 35°), whereas the minimum mid-inner wing *ψ* is at around 50°. Between *t*/T = 0.8 and the end of the downstroke at *t*/T = 1.0, the wing undergoes rapid rotation, and it is during this time that the outer wing undergoes nearly 60° of rotation whereas the inner wing rotates by about 40°. Also from Fig 9(B), it is apparent that the wing undergoes advance rotation, i.e. the wing starts to pitch upwards before the end of the downstroke to optimize the upstroke motion, and starts to pitch downwards during the upstroke for a more effective downstroke. As will be shown later, wing twist has a significant effect on lift generation.

#### Cambering (chordwise)

When chordwise cambering is included in the kinematics, the modeled equation describing wing motion is:

$$W(t=t_{n}){=W}_{i,j,n}=R_{n}(\phi )R_{n}(\theta )\begin{array}{c} R_{i,n}(\psi )[P_{i,j}+C_{i,j,n}] \end{array}$$ (5)

Here, the equation is nearly identical to the pitching motion, except that [**P**_i,j_+**C**_i,j,n_] represents a cambered airfoil instead of the previous chord line (**P**_i,j_), and the camber varies with time. This is an additional degree of flexibility featured in the wing.

**Fig 10:**
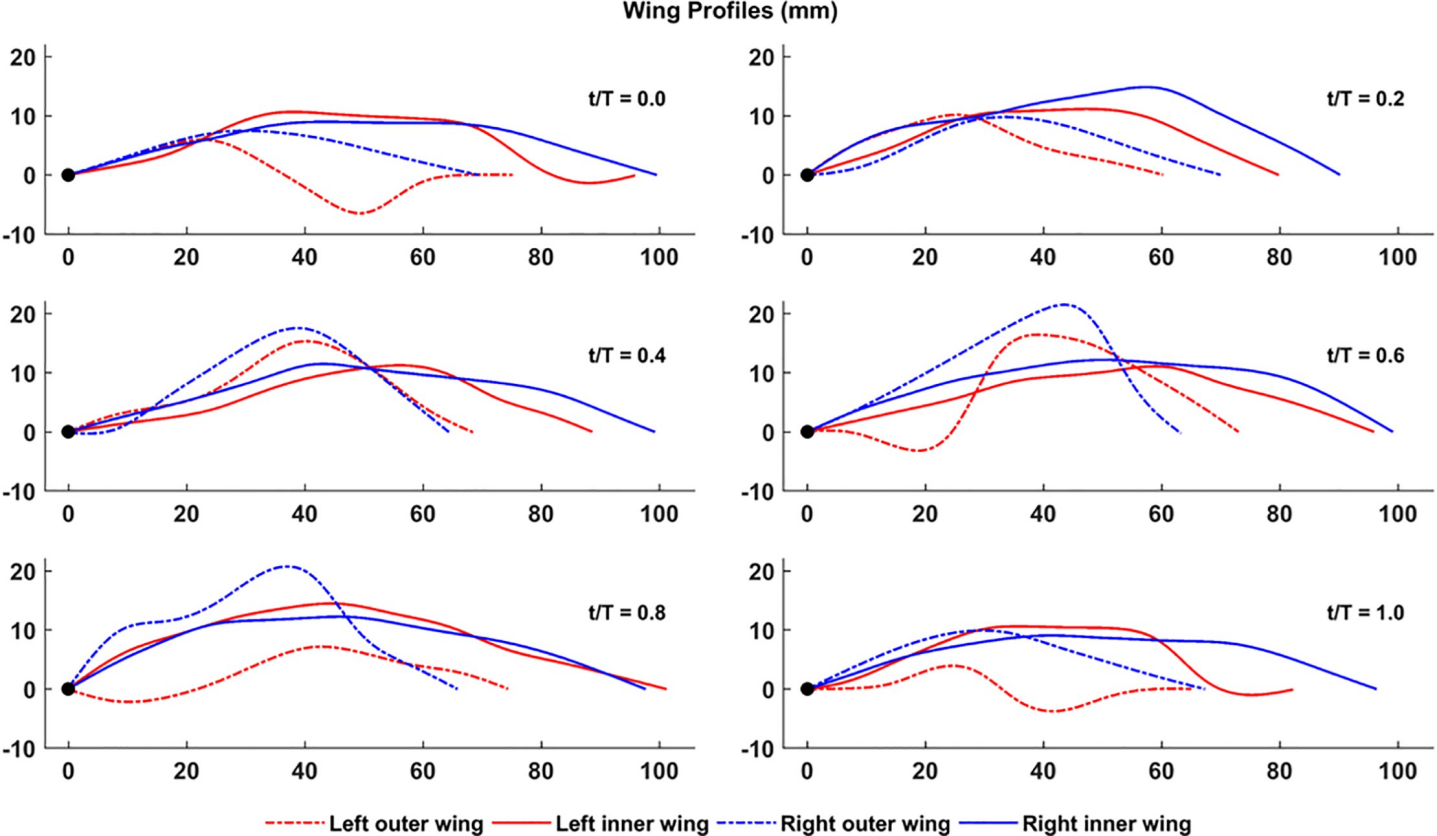
Temporal evolution of airfoil shape at mid-inner and outer sections on both wings over one flapping cycle (53 ms to 182 ms).

Fig 10 shows the temporal evolution of the shapes of four different airfoil sections on both wings, at the mid-inner and mid-outer wing locations defined in Fig 8. The leading edges are identified using a black dot at the (0,0) location in all the plots and both axes are in mm. During the downstroke (*t*/T = 0.6 to 0.8), the inner wing section exhibits a maximum chord length of approximately 100mm, whereas during the upstroke, the chord decreases to between 80mm and 90mm. The outer wing section, on the other hand, maintains a fairly constant chord length between 60mm and 70mm. There is a marked difference in the behavior of the inner wing and the outer wing sections on both wings: the variability in airfoil shape during the flapping cycle is significantly more for the outer wing sections, exhibiting a variety of shapes and much larger cambers than the inner wing section. The airfoil cutting planes in the outer wing pass through phalanges across different digits, which could alter airfoil camber considerably. The inner wing, on the other hand, assumes shapes more in line with conventional airfoils except at the start of the upstroke (*t*/T = 0 and 1.0). Here, the left wing shows a clear inflection point at 70–80% chord length, which is absent on the right wing. The outer wing sections on both wings behave rather independently, showing large variance in the camber values through the flapping cycle. The right outer wing shows a relatively consistent trend of having peak camber around the mid-chord, while the left wing behavior is more complex. These complex shapes can be attributed to the flexible membrane wing that stretches and relaxes during the flapping cycle depending on the aerodynamic and inertial forces acting on it.

**Fig 11:**
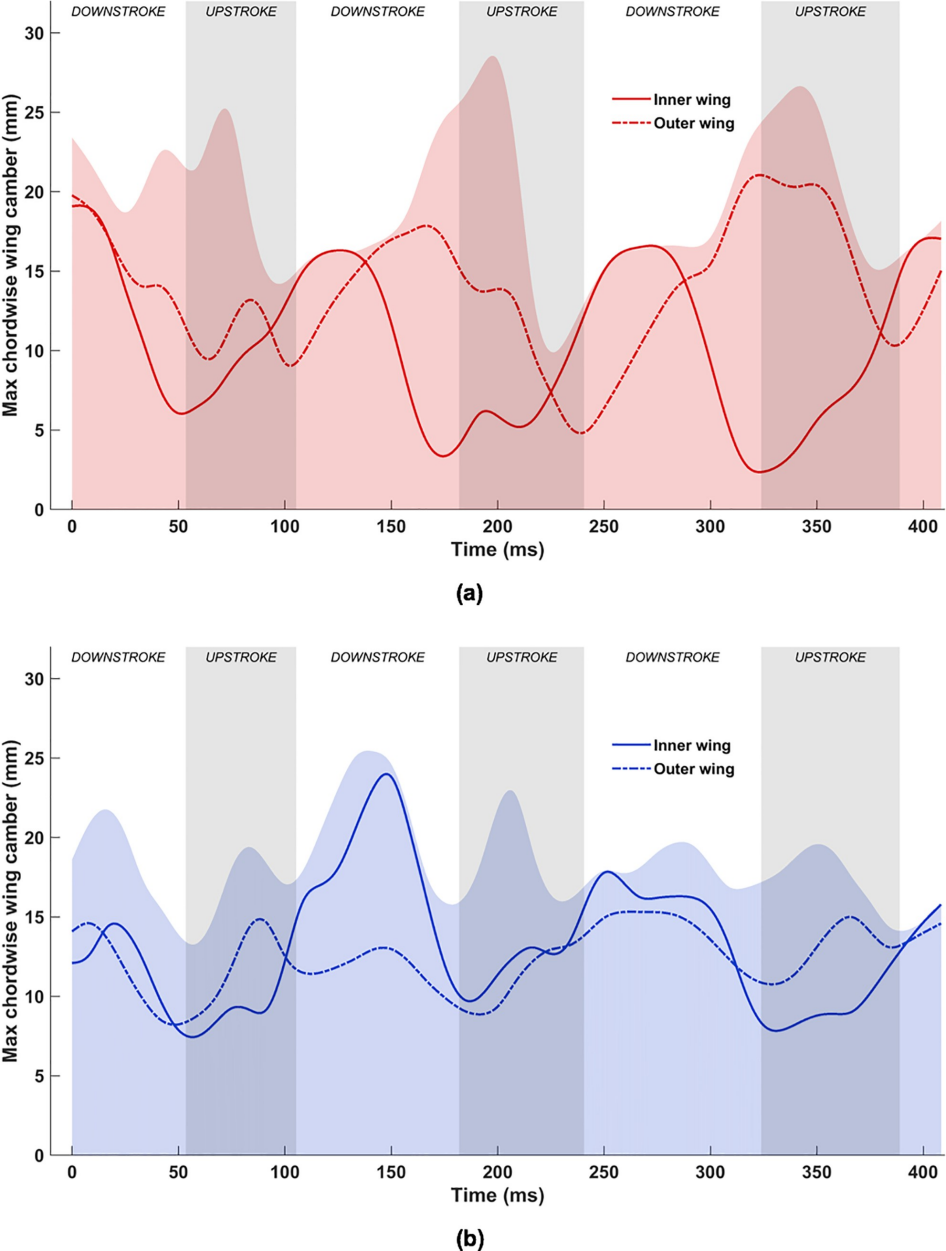
Temporal evolution of chordwise camber at different airfoil sections along the span: (a) left wing, (b) right wing.

In Fig 11, the time evolution of the maximum absolute chordwise camber over the measured flight at all span locations are shown as shaded regions. The lines (solid and dashed) are the maximum camber at the highlighted mid-sections of the inner and outer wing from Fig 8. Evidently, there is a wide spread in the maximum camber, which demonstrates the flexible nature of the wing, and the ability of bats to actively control wing morphology, as well as respond to both aerodynamic and inertial forces. For example, near the middle of the 2nd upstroke (≈200 ms), the maximum camber on the left wing reaches almost 29 mm and on the right wing, it reaches a local maximum of 23 mm. These represent deflections that are approximately 40% and 30% of the standard mean chord (74 mm).

Temporally, the right wing shows a more consistent cambering trend between the inner and outer wing: except for out-of-sync cambering during the 2^nd^ downstroke, both the inner and outer right wing follow the same cadence. The left wing also starts with a similar consistent cambering trend, but during and after the 2^nd^ downstroke, the inner and outer left wing exhibit out of phase maximum cambering. Over the measured kinematics, the mean maximum camber over the full span of the wing is between 17 mm and 20 mm, which is approximately 25% of the standard mean chord.

#### Flexion

Incorporating wing flexion completes the integration of all the movements of bat wing kinematics. The modeled equation is the same as shown earlier (Eq 1):

$$W\left({t=t}_{n}\right){=W}_{i,j,n}{=R}_{n}{(\phi )R}_{n}{(\theta )R}_{i,n}(\psi )\left[P_{i,j}{+C}_{i,j,n}{+D}_{i,n}\right]$$

Here, **D**_i,n_ represents the time dependent displacement of each airfoil about its spanwise location, and this is allowed along all three axes.

**Fig 12:**
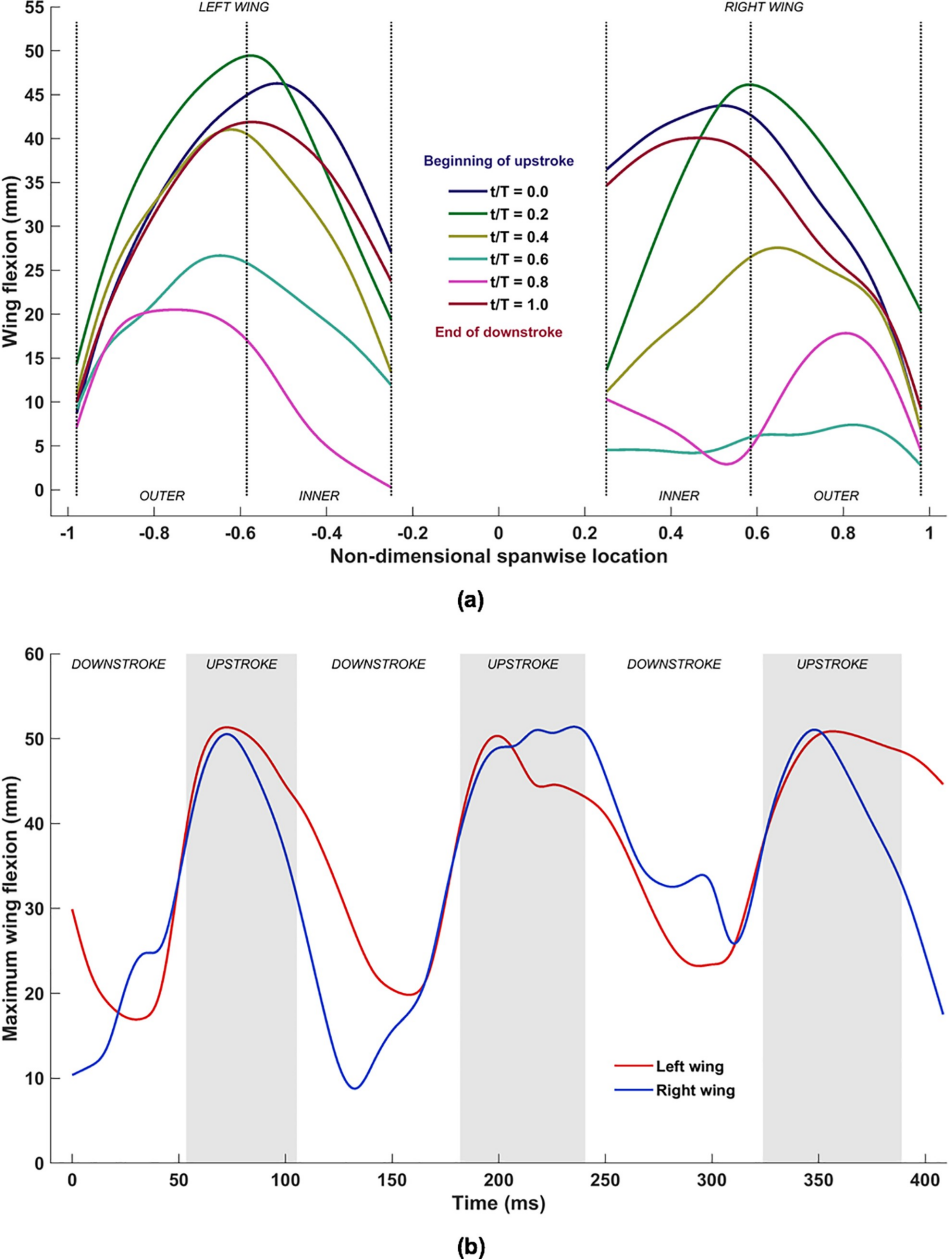
(a) Spanwise variation of flexion over one flapping cycle (53ms to 182ms), (b) temporal evolution of maximum flexion.

Fig 12(A) shows the spanwise evolution of flexion of both wings during a single flapping cycle (53ms to 182ms) in terms of the off-span displacement: $|D_{i,n}|={\left(x_{i,n}^{2}+z_{i,n}^{2}\right)}^{1/2}$. The high variability and the out-of-phase morphology between the left and right wings is testament to both the highly articulated nature of the wings and the inherent asymmetry in natural flight. It is noteworthy that the majority of flexion occurs towards the end of the downstroke (*t*/T = 0 and 1.0) and during the early part of the upstroke (*t*/T = 0.2), during which the bat retracts both wingtips towards the body, resulting in maximum flexion (≈50mm at *t*/T = 0.2) falling around the wrist on each wing. There is a marked reduction in flexion during the downstroke, when the bat extends the wing outwards to maximize the projected area, in addition to optimizing the planform using the pitch angle distribution (Fig 9(A)).

Fig 12(B) shows the temporal evolution of the maximum flexion of both wings during all three flapping cycles. The same trend of higher flexion magnitudes during the upstroke to reduce the planform area, and relatively lower magnitudes during the downstroke is evident throughout the flight path. Differences between the left and right wing flexion is further evidence that the bat uses multiple movements to optimize and effect even a simple near-straight and level flight. The mean maximum flexion over the three flapping cycles is approximately 35mm, which is ≈50% of the standard mean chord.

## Aerodynamic analyses

The investigation into the cumulative impact of each physical movement on force production is presented here. The analysis first considers the basic flapping motion, and builds up complexity progressively by including additional movements to construct the complete native wing kinematics. Throughout this process, insight is gained into the effect of each motion on the unsteady flow field, and on force production.

### Simulations setup

All simulations were performed using an in-house developed software, GenIDLEST [56] (Generalized Incompressible Direct and Large Eddy Simulation of Turbulence). It is a parallelized code developed for time-dependent fluid flow calculations that solves the incompressible Navier-Stokes equations in a generalized coordinate framework. Details about the formulation can be found in Ref. [57].

#### Governing equations and methods

The nondimensional Navier-Stokes equations for incompressible flows in a generalized coordinate framework are:

$$\frac{\partial u_{i}}{\partial x_{i}}=0$$ (6)

$$\frac{\partial u_{i}}{\partial t}+\frac{\partial }{\partial x_{j}}(u_{i}u_{j})=-\frac{\partial p}{\partial x_{i}}+\frac{\partial }{{\partial x}_{j}}\left[\frac{1}{Re}\frac{\partial u_{i}}{\partial x_{j}}\right]$$ (7)

where the state variables are nondimensionalized as:

$$x_{i}=\frac{x_{i}^{*}}{L_{ref}^{*}};u_{i}=\frac{u_{i}^{*}}{u_{ref}^{*}};t=\frac{t^{*}u_{ref}^{*}}{L_{ref}^{*}};p=\frac{p^{*}-p_{ref}^{*}}{\rho_{ref}^{*}{u_{ref}^{*}}^{2}};Re=\frac{\rho_{ref}^{*}u_{ref}^{*}L_{ref}^{*}}{\mu_{ref}^{*}}$$

**Fig 13:**
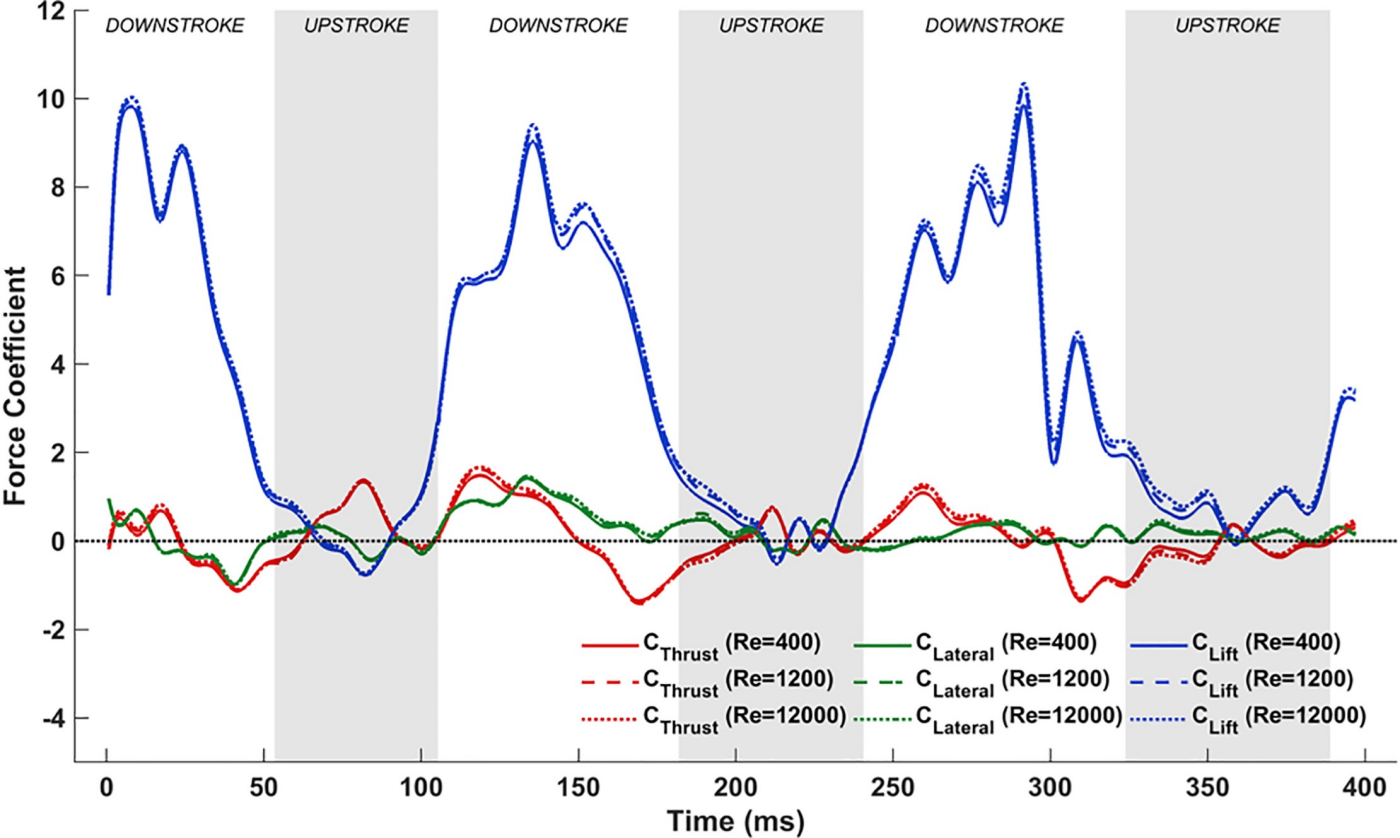
Comparison of force coefficients during bat flight (native kinematics) at three different Reynolds numbers (400, 1,200 and 12,000).

Here, the superscript ‘*’ and the reference parameters are dimensional quantities, and subscripts ‘*i*’ and ‘*j*’ are indices according to the Einstein summation convention. In the present work, the standard mean chord ${c=L}_{ref}^{*}$ = 0.074 m, and $u_{ref}^{*}$ = 2.57 m/s is the average flight velocity. This standard mean chord is defined as the ratio of the maximum planform area of the bat to the tip-to-tip wingspan. Nondimensional forces coefficients are normalized as $C=F^{*}/\frac{1}{2}\rho_{ref}^{*}u_{ref}^{*}S^{*}$, where F* is the dimensional force component, $\rho_{ref}^{*}$ = 1.2 kg/m^3^ is the reference ambient air density, $u_{ref}^{*}$ is the reference freestream velocity, and S* = 0.0385 m^2^ is the maximum planform area of the wing during the downstroke. Based on these conditions, the nominal flight Re ≈ 12,000. In a previous work [47], it was shown that the nondimensional forces (lift, thrust, etc.) are independent of the Reynolds number for the unsteady separated vortex dominated flows encountered, and noted that the flow structures are more coherent and amenable to physical interpretation at lower Re. Such clarity augments the analysis of flow features that develop from different combinations of movements, more so while establishing the paradigm for decomposing complex kinematics of flapping flight. Thus, simulations were conducted at an arbitrary low Re = 400. Evidence of the insensitivity of nondimensional forces to Reynolds number is presented in the form of comparisons of various computed force components at Re = 12,000, 1,200 and 400 in Fig 13. Additionally, supporting information S5 Video, S6 Video, and S7 Video show a comparison between Re = 400, Re = 1,200, and Re = 12,000.

The governing equations are solved using a finite-volume procedure with a second-order central difference operator used for the convection and diffusion terms. Linear systems resulting in the implicit treatment of the momentum equations and the solution of the elliptic pressure equation are solved using a preconditioned BiCGSTAB method.

#### Computational domain and boundary conditions

**Fig 14:**
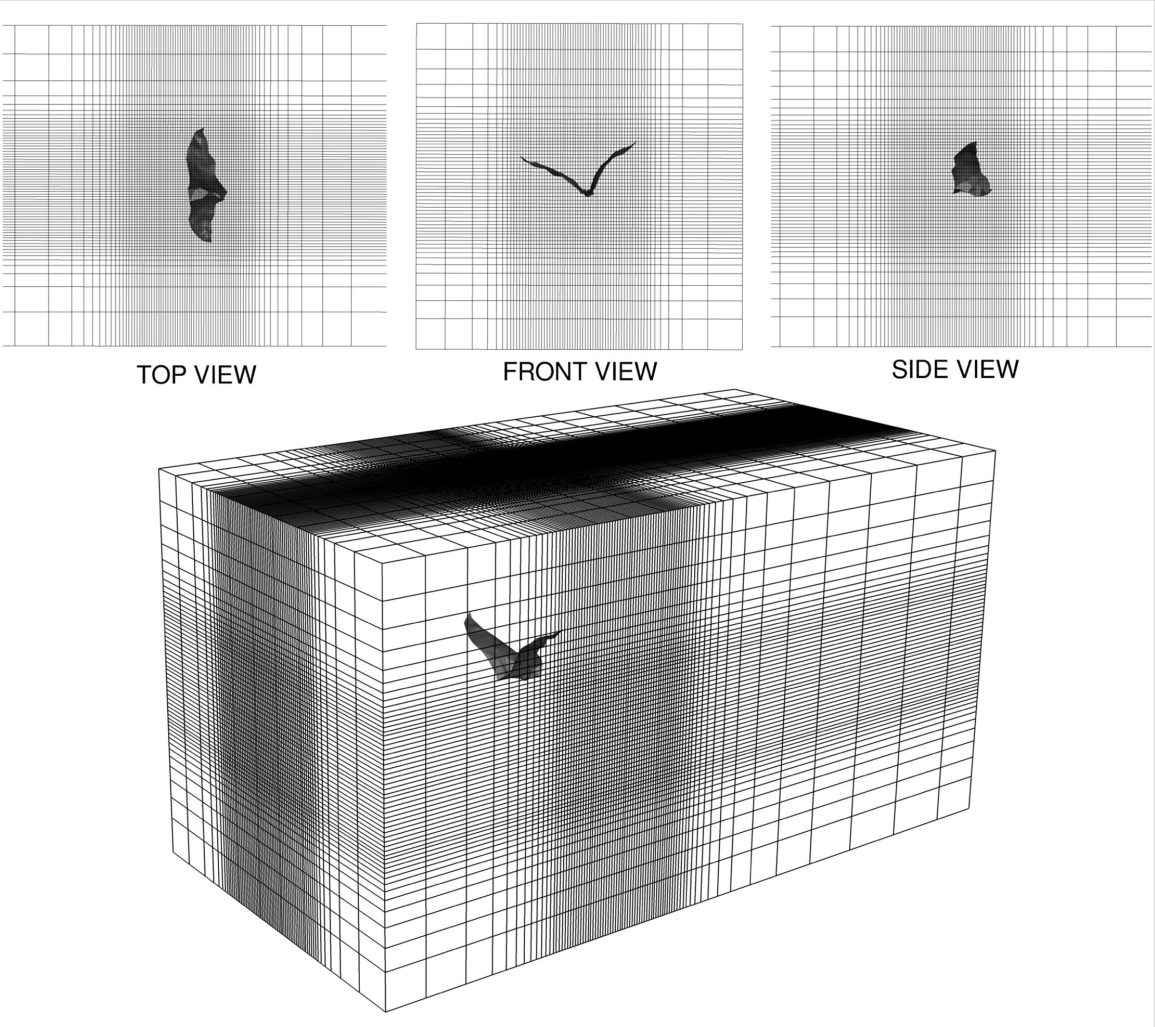
Perspective view of background mesh (plotting every 5th grid line of the 30*c* × 16*c* × 16*c* domain) for fluid simulation enclosing the bat, with orthographic projections of top (18*c* × 16*c*), front (16*c* × 16*c*) and side (18*c* × 16*c*) views. The total cell count was 32 million.

The computational domain, shown in Fig 14, consists of a rectangular region that represents the experimental flight tunnel (1.18 m × 1.18 m cross-section). Relative to the standard mean chord length, the domain extends 30*c* × 16*c* × 16*c* along the streamwise, lateral and vertical directions, respectively. Along the streamwise direction, from the inlet to the outlet, the background mesh is divided into three sections: (i) a relatively coarse section that extends 7*c* upstream, (ii) a refined section around the bat geometry to resolve flow around the wings that is 7*c* long, and (iii) a long coarsened section leading to the outlet that extends 16*c* downstream. A similar strategy was applied to the lateral and vertical directions. The simulation was run using a moving reference frame with an equal and opposite velocity to the nominal flight velocity. This allows the computational domain to follow the bat over a long flight path while only requiring a short computational domain.

The bat wing was represented as a moving immersed surface in a volumetric background mesh using an indirect-forcing sharp-interface IBM for infinitesimally thin surfaces. The wing was defined by a triangulated surface mesh. For the wing in motion, the fluid-solid interface was tracked in the flow at each time step based on the prescribed wing kinematics. The IBM procedure and implementation of boundary conditions are described in detail in Nagendra et al. [58]. The method was previously applied by Viswanath et al. [47,55] to bat flight, and by He and Tafti [59,60] to force and heat transfer calculations in particle suspensions. Most recently it was also applied by Windes et al. [52] to the native kinematics of the *H*. *pratti* in level flight.

The moving frame of reference was imposed using a constant inlet air velocity of 2.57 m/s and a sidewall slip velocity equal to the inlet velocity. The relative inlet and relative wall velocity of 2.57 m/s corresponds to zero velocity in a ground frame of reference. A zero-gradient condition was specified at the outlet of the domain noting that during the 0.42s of simulation time, the wake only extends up to 10*c* downstream of the bat and does not reach the outlet plane. The time-steps for all the simulations were approximately in the range of 25μs.

In all the cases presented, the wing surface was defined by approximately 70,000 triangular surface elements with a mean side of 1.26 mm. A grid independence study was carried out using four different Cartesian background meshes, ranging from 20-million to 70-million computational cells, while keeping the wing surface mesh the same. The results are tabulated in Table 1. The averaged differences (*E*_*i*_) of the forces (*F*_*i*_: net thrust, lateral force and lift) from different meshes were calculated using:

$$E_{i}=\frac{1}{N}\sum^{N}\frac{{|F}_{i,n}-{\hat{F}}_{i,n}|}{\left|{\hat{F}}_{i,n}\right|}$$ (8)

Here, ‘*i*’ represents different directional components of force, and ‘*n*’ is the time-step. ${\hat{F}}_{n}$ is the baseline force calculated on the 70-million-cell background mesh. Estimated forces were grid independent for all meshes tested, with a maximum difference of 1.6% in the lateral force for the 20-million-cell background mesh. Related grid independency studies for the same flight of the *H*. *pratti* with native wing kinematics can be found in Windes et al. [52]. In the same study, dynamical analysis was provided to validate the calculated forces on the bat. There, it was shown that a trajectory predicted from the computed fluid forces matched well with the experimental flight trajectory.

**Table 1:** Grid independence study.

****Total number of cells****	****Length of cell in refined region****	****Aspect ratio of cell in refined region**** (Δx: Δy: Δz)	*E*_1_****(Net thrust)****	*E*_2_****(Lateral)****	*E*_3_****(Lift)****
20×10^6^	*c*/28	1 : 1 : 1	0.05%	1.6%	0.27%
32×10^6^	*c*/36	1 : 1 : 1.5	0.03%	1.2%	0.05%
45×10^6^	*c*/36	1 : 1 : 1	0.01%	1.1%	0.05%
70×10^6^	*c*/50	1 : 1 : 1	-	-	-

Based on the results from this study, all simulations presented hereafter were performed on the 32-million-cell background mesh. A 3D representation of the grid showing every fifth cell is provided in Fig 14. The vortical structures in proximity to the wings, which directly impact force production, are well resolved in the refined region of the grid as shown in supporting S8 Video. Each was run on 120 Intel Broadwell processors at Virginia Tech’s Advanced Research Computing facility, and took approximately 40hrs of wall clock time to complete the measured flight path of the bat.

### Aerodynamics simulation results

Table 2 summarizes the time-averaged aerodynamic force coefficients from the simulations—net thrust is defined as the resultant forward force, lift is the net vertical force, and the lateral force is the third force component perpendicular to thrust and lift. It is evident that the flapping motion by itself produces a modest lift, and induces drag. The inclusion of stroke plane deviation increases lift by more than 40%, and more importantly, balances out the drag. The pitching motion further increases the mean lift by nearly 165%, and produces a net thrust force. Chordwise camber reduces net thrust by 20%, but increases lift by nearly 35%. Finally, the integration of all movements maximizes the lift force, reduces the net thrust to a modest value, and imparts a lateral force on the bat. These are in agreement with the small acceleration recorded during bat flight, and the nominal lateral movement off the straight flight path. Dynamic analysis using the native kinematics performed in Windes et al. [52] using the predicted instantaneous forces shows good agreement with the measured trajectory of the bat. Flight path animations showing the wing kinematics and isosurfaces of coherent vorticity are included as Supporting information (See S1 Video, S2 Video, S3 Video, S4 Video, S5 Video).

**Table 2:** Time-averaged forces over two flapping cycles (53ms to 324ms).

****Forces****	****I****	****I+II****	****I+II+III****	****I+II+III+IV****	****I+II+III+IV+V****
${\bar{C}}_{Thrust}$	-0.23	0.00	1.23	0.97	0.13
${\bar{C}}_{Lateral}$	-0.01	0.00	0.02	0.09	0.25
${\bar{C}}_{Lift}$	0.61	0.87	2.30	3.09	3.47

(I–Flapping, II–Stroke plane deviation, III–Pitching, IV–Cambering, V–Flexion)

**Fig 15:**
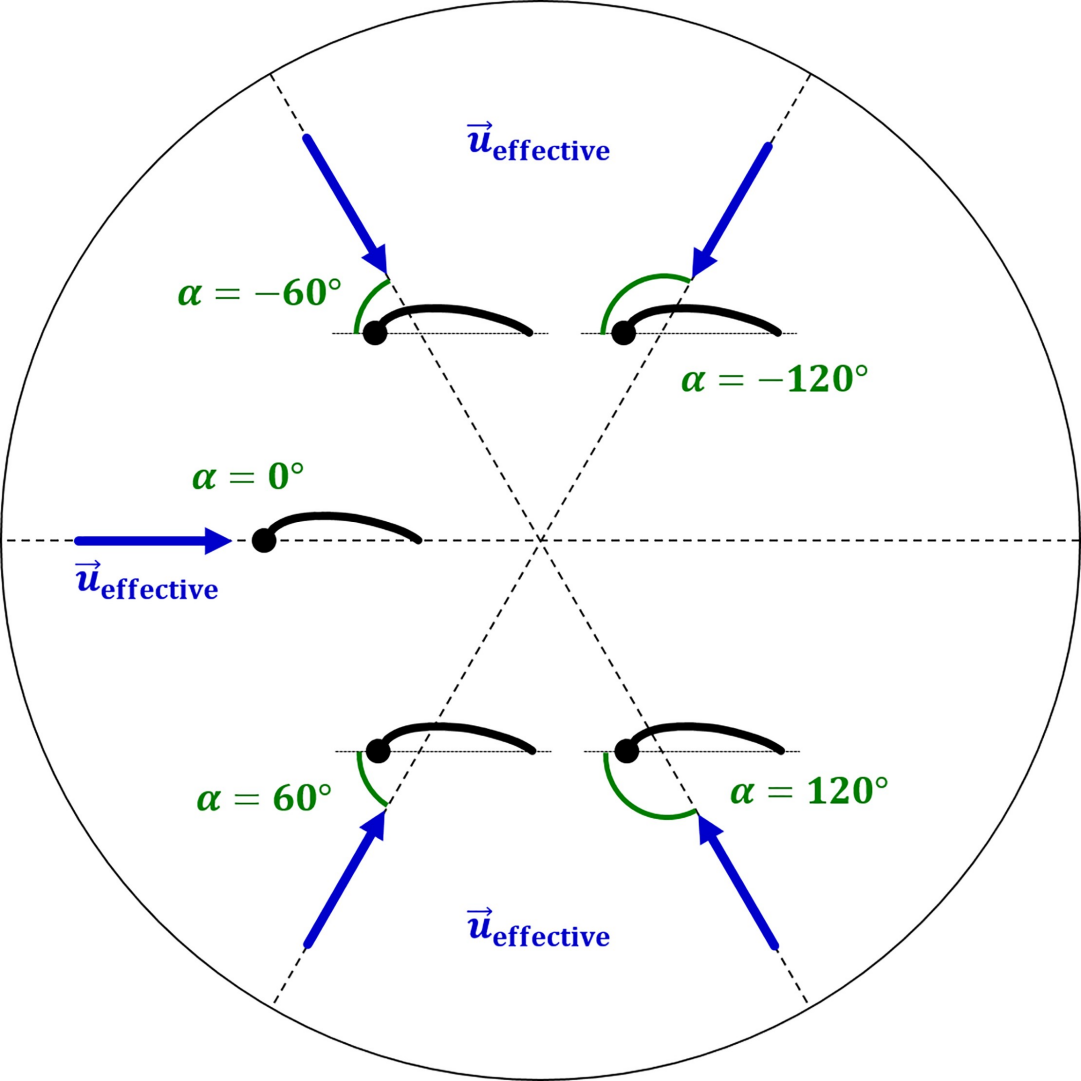
Interpretation of *α*_effective_ (or *α*).

The following discussions elaborate on the unsteady flow physics that effects the results in Table 2. To interpret aerodynamic lift, which is the primary force of interest in the nominally straight and level flight of the *H*. *pratti*, an effective angle of attack (*α*_effective_) is defined. As will be shown, there is a strong correlation between the instantaneous lift force and the instantaneous effective angle of attack that allows for a distinctive interpretation of lift force production across different movements. The effective angles of attack are the angles that the local relative flow vector makes with each chord line of an airfoil section across the span of the wings. Thus, *α*_effective_ varies with time and along the span of each wing. The relative flow vector is the vector sum of the instantaneous forward flight velocity of the bat and the instantaneous velocity of the leading edge imposed by the kinematics in the stroke plane. A schematic interpretation of *α*_effective_ is shown in Fig 15. Similar to static airfoil theory, positive angles are indicative of positive lift production, whereas negative angles are detrimental and produce negative lift.

Using the aforementioned combinations of wing movements, the overall native kinematics of the wings are re-composed sequentially, from the simplest to the most complex and complete, and the associated aerodynamics are simulated and analyzed.

#### Flapping motion

**Fig 16:**
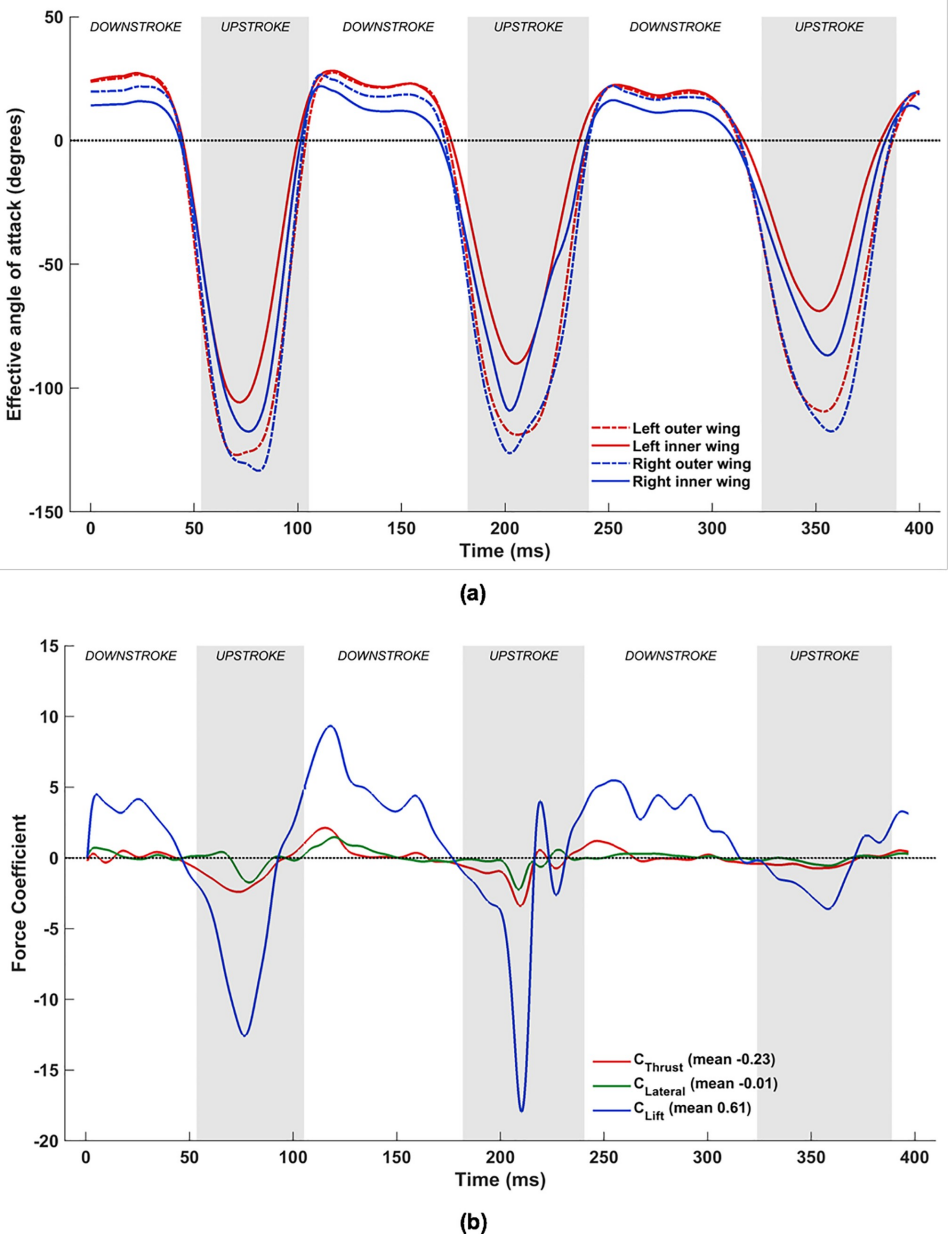
Flapping motion: (a) Time variation of *α*_effective_, (b) Forces evolution averaged over two flapping cycles (53ms to 324ms).

Fig 16(A) shows the effective angle of attack (*α*_effective_) at the previously defined mid-inner and mid-outer wing sections, and Fig 16(B) is the plot of the simulated aerodynamic forces when flapping motion alone is included in the wing kinematics.

The effective angle of attack, *α*_effective_, can be used to explain the generation of lift for a large part of the flapping cycle by associating Fig 16(A) with Fig 16(B). During the downstroke, most of the airfoil sections (chord lines here) experience a positive *α*_effective_, and, thus, generate lift. Conversely, during the upstroke, *α*_effective_ is mostly negative, and results in a negative lift. During a brief time interval in the 2^nd^ upstroke, between ≈215 ms and ≈225 ms, the aerodynamics show evidence of positive lift. Fig 6(B) provides some clues to this phenomenon. It shows that during the initial part of the upstroke, the right wing flapping angle increases rapidly at first until about 215 ms, followed by a sudden decrease in the rate of change. This is also reflected in the inflections in the *α*_effective_ curve in Fig 16(A), at 215ms on the outer wing and at 225ms on the inner wing. The faster upward movement of the right wing followed by the slow down creates a transient LEV on the dorsal surface of the wing resulting in lift production until the LEV dissipates away. This phenomenon is evident only in the second cycle. The averaged forces presented in Fig 16(B) reveal that merely flapping in the stroke plane (*β* = 53°) induces a net drag on the bat, and a modest lift (${\bar{C}}_{Lift}$ = 0.61).

**Fig 17:**
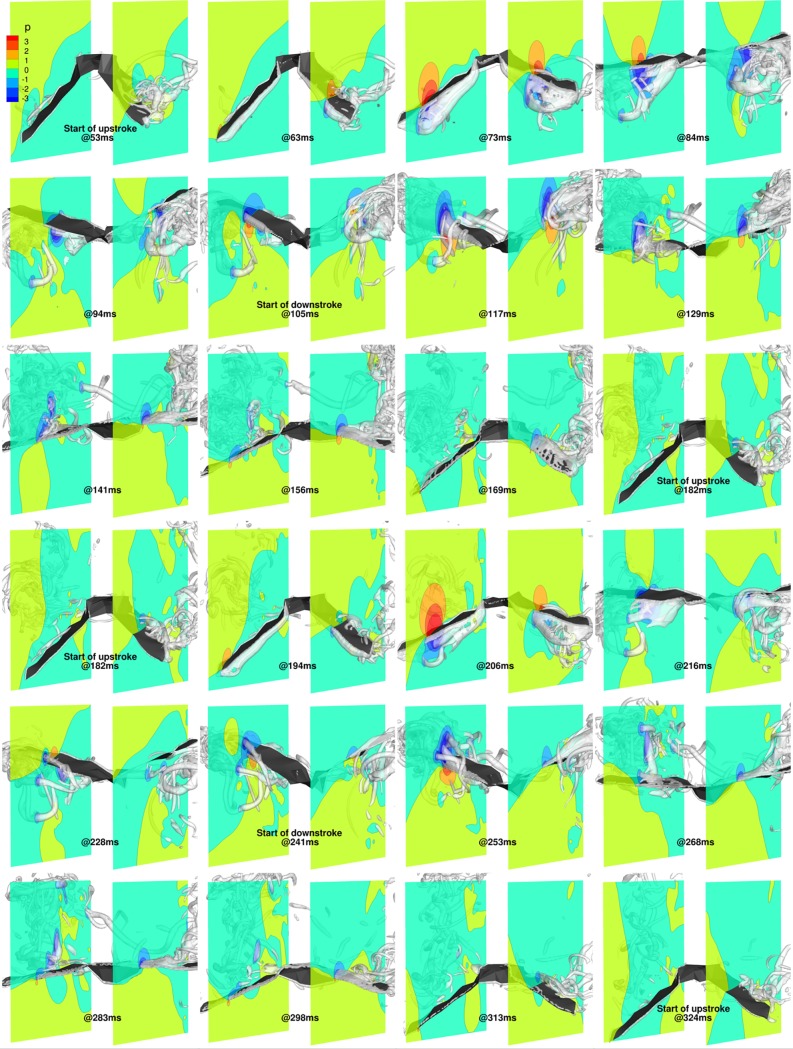
Flapping motion (53 ms to 324 ms): 3D view of wing with pressure contours at y/c = 1.75 and y/c = -2, and isosurfaces of coherent vorticity (***ω***_***c***_ = 5).

In order to understand the flow field in more detail, Fig 17 shows the 3D wings overlaid with isosurfaces of coherent vorticity, and nondimensional pressure contours on cutting planes positioned at *y*/*c* = 1.75 and *y*/*c* = -2 (approximate wrist locations of the fully stretched right and left wings, respectively) for two complete flapping cycles, from 53 ms to 324 ms. The coherent vorticity is educed from the flow field using the complex eigenvalues of the strain rate tensor [61]. Evidently, regions of low pressure are associated with coherent vortex cores that have a direct bearing on force generation on the wing surface.

At 53 ms, during the start of the upstroke, remnants of LEVs and tip vortices generated on the outer wings during the previous downstroke are still present in the wake. During the upstroke, by 63 ms, due to the negative *α*_effective_, LEVs form on the ventral side of the wings, which grow in strength until about 73 ms, when they detach from the surface and shed. During this period, *α*_effective_ extends its negative value, and the LEVs gather strength and increase the pressure differential between the dorsal and ventral sides of the wings, augmenting the negative lift. Subsequently, with the shedding of the first LEVs, weaker LEVs form on the ventral side, maintaining the negative lift, albeit of smaller magnitude. During the transition from upstroke to downstroke, beyond 94 ms, as *α*_effective_ becomes positive, the weaker LEVs separate from the ventral side, with the formation of a new set of LEVs on the dorsal surface of the wings. By 105 ms, the dorsal LEVs are firmly established, resulting in low-pressure regions on the upper wing surface and a positive lift force.

During the downstroke, which started at 105 ms, the LEVs on the dorsal side of the wing grow in strength and start to detach from the outer wing, as inferred from the vorticity isosurfaces at 129 ms. Soon after separation, a second set of LEVs start to form at the leading edge on the dorsal side of both wings, which are evident at 141 ms and at 156 ms. Towards the end of the downstroke, by around 169 ms, these LEVs have separated and the wings are ready to begin the upstroke at 182 ms with the formation of a new set of LEVs on the ventral side.

A similar overall trend of LEVs being formed alternatively on the ventral side during the upstroke and the dorsal side during the downstroke is evident during the 2^nd^ flapping cycle (182 ms to 324 ms). The only notable difference starts at around 206 ms when the LEV from the ventral side appears to have been shed earlier than during the previous flapping cycle. Subsequently, at 216 ms, pressure contours on the right wing are quite different from the corresponding time-stamp in the 1^st^ flapping cycle (84 ms). Whereas a clear pressure differential is established at 84 ms, which results in a consistent negative lift during this phase, at 216 ms the wing is enveloped by a low pressure bubble that gives way to a non-uniform pressure distribution at 228 ms. Here, the leading edge of the wing sees a low pressure region on the dorsal side, while the trailing edge sees a high pressure bubble. This uneven distribution is attributed to the acceleration and deceleration of the right wing, resulting in the weak inflection points in the *α*_effective_ curves.

The upstroke produces most of the negative lift, which is a direct result of negative *α*_effective_ throughout the span. Similarly, the positive *α*_effective_ produces positive lift during the downstroke. More specifically, the negative lift peak at around 74 ms is a result of the LEVs growing and subsequently separating from the surface. Similarly, the positive peak lift at around 115 ms is a result of the maximum strength of the LEVs on the dorsal side of the wing. These values are more inconsistent during the 2^nd^ flapping cycle due to the spasmodic nature of the right wing kinematics, which leads to a less orderly behavior of the LEV. For thrust production, as the wing maintains the same pitch angle distribution throughout the flapping cycles (refer Fig 5), net thrust and lift are strongly correlated. As the wing starts with a slightly pitched down orientation and retains the pitch angle distribution throughout the flapping cycles, lift and net thrust are positively correlated, as seen in Fig 16(B).

#### Combined flapping and stroke plane deviation

**Fig 18:**
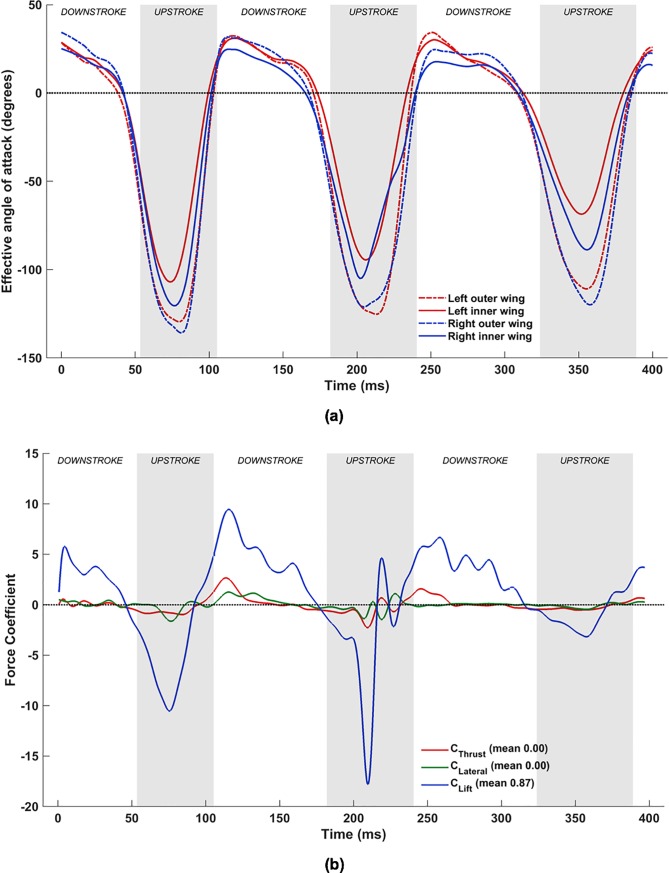
Combined flapping and stroke plane deviation: (a) Time variation of *α*_effective_, (b) Forces evolution averaged over two flapping cycles (53 ms to 324 ms).

**Fig 19:**
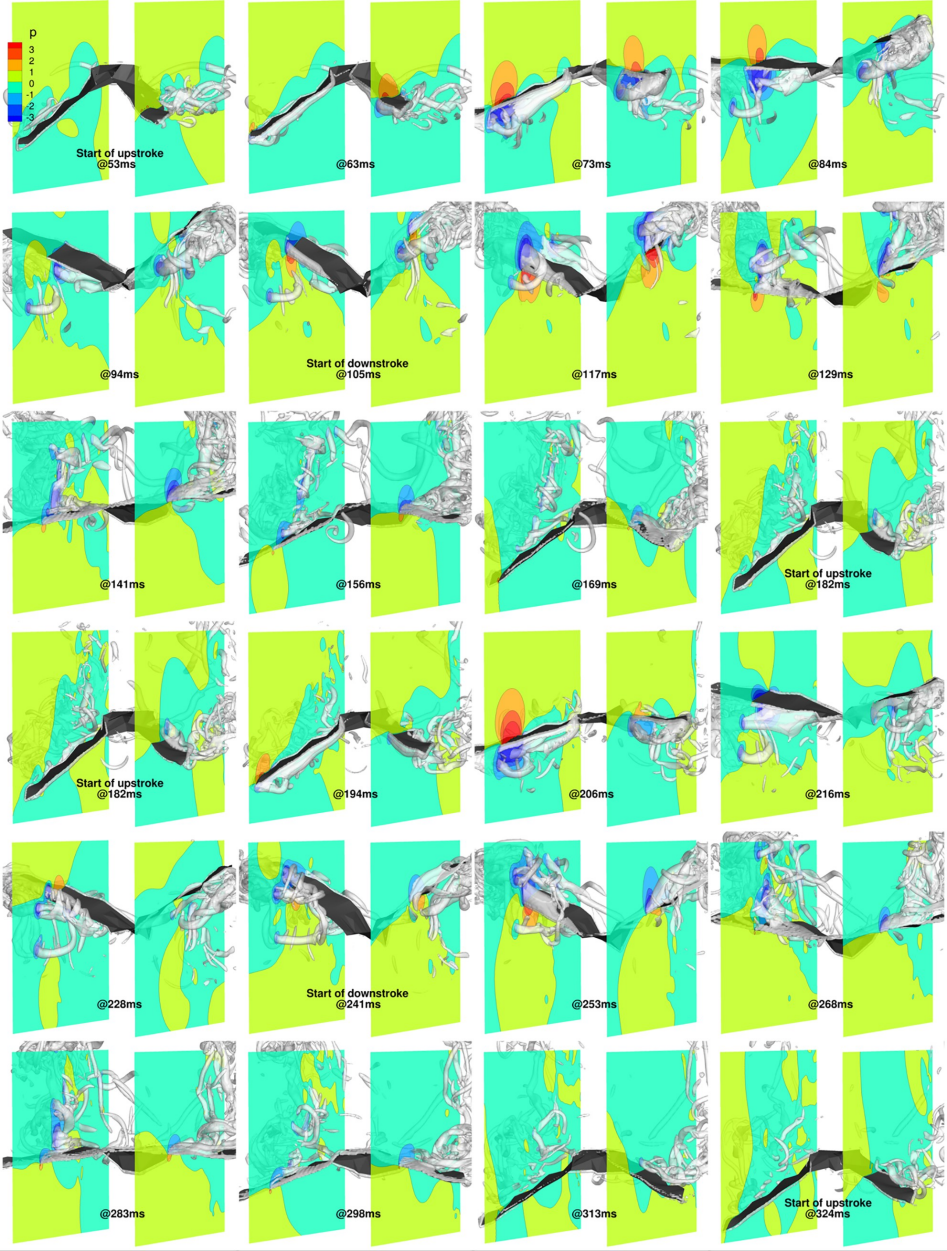
Combined flapping and stroke plane deviation (53 ms to 324 ms): 3D view of wing with pressure contours at y/c = 1.75 and y/c = -2, and isosurfaces of coherent vorticity (***ω***_***c***_ = 5).

Combining stroke plane deviation with the flapping motion does not have a significant impact on *α*_effective_, as evident when comparing Fig 18(A) with Fig 16(A). As a result, the underlying unsteady vorticity dynamics is only marginally affected by the inclusion of this motion (Fig 19 vs Fig 17). The noteworthy difference between the *α*_effective_ plots is at the start of each downstroke: whereas the flapping motion maintains a near constant *α*_effective_ during this phase, inclusion of *θ* (stroke plane deviation angle) into the kinematics results in a non-insignificant increase in *α*_effective_ at the beginning of each downstroke. This is most prominent on the left wing during the 3^rd^ downstroke (≈250 ms).

A more subtle difference is apparent during the 1^st^ upstroke when comparing Fig 18(A) with Fig 16(A): between 73 ms and 84 ms, *α*_effective_ on the left outer-wing reaches the local minimum 10 ms later when compared to the flapping-only case, i.e., there is a more gradual attainment of the peak negative value when *θ* is included in the kinematics. This delay of ≈10 ms results in a weaker LEV and tip vortex on the left wing (Fig 17 and Fig 19, at 84 ms), which in turn reduces the magnitude of the peak negative lift during this phase of flight. This is also evident in the pressure contours at this time instant: whereas the low pressure on the ventral side of the left wing remains substantial for the flapping motion, when *θ* is added to the kinematics, a smaller low pressure bubble forms on the ventral side of the left wing. This is also the same time-period when *θ* changes direction for the left wing, after reaching a peak deviation of ≈ 25° off the stroke plane (Fig 7(B)). The combined effect of the movements results in a nearly 25% reduction in the peak negative lift. On the other side, the right wing maintains a comparatively steady *θ* ≈ 15° deviation during this time period, and exhibits a similar pressure signature as the case without *θ*.

During the 2^nd^ upstroke, the right wing stroke plane deviation increases from ≈7° to about 12°, while the left wing *θ* decreases from ≈17° at the start of the stroke to 10° by the end of it. The out-of-phase stroke plane deviation between the left and right wings and the spasmodic nature of the kinematics during the 2^nd^ upstroke seem to mitigate differences in the flow, and a similar clear distinction in lift is not evident during a similar stage of this stroke (at 216 ms). When integrated over the two flapping cycles, the cumulative impact of the differences during the upstroke and downstroke result in a noteworthy 40% increase in the average lift force, highlighting the impact of fine-tuned movements of the bat wing. The delicate movement of the left outer-wing during the 1^st^ upstroke also reduces the peak negative net thrust during that time-period, and cumulatively nullifies the mean drag that was computed for the flapping-only simulation.

#### Combined flapping, stroke plane deviation and pitching

**Fig 20:**
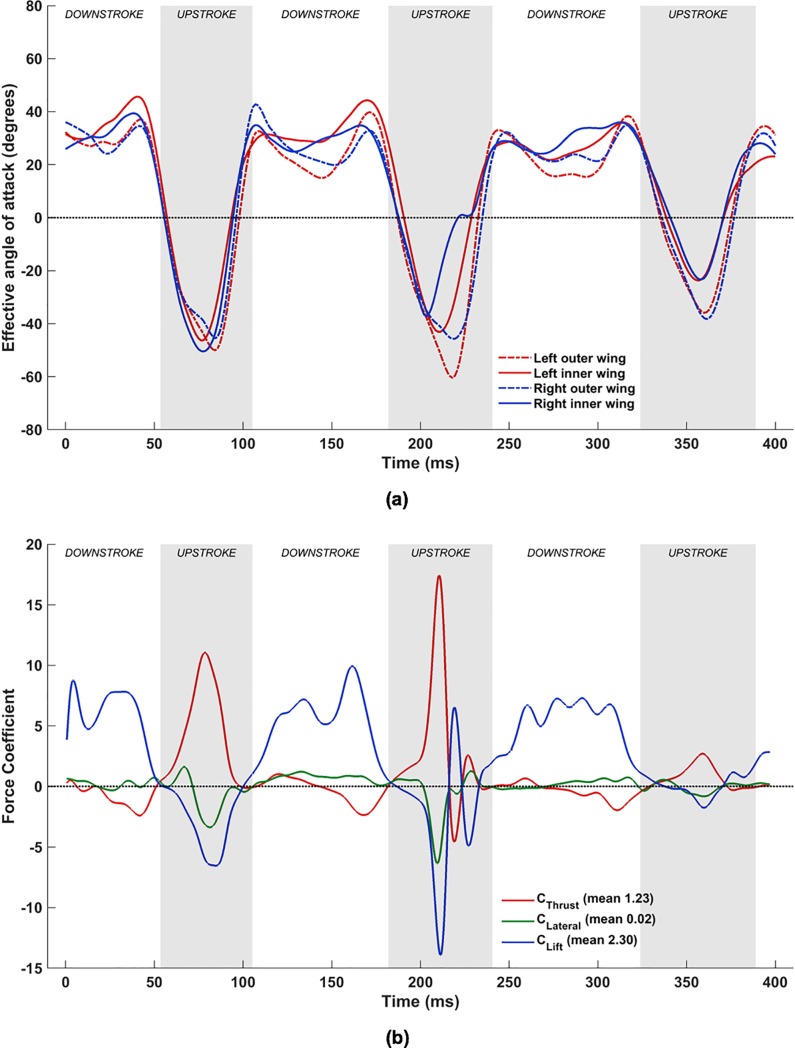
Combined flapping, stroke plane deviation and pitching: (a) Time variation of *α*_effective_, (b) Forces evolution averaged over two flapping cycles (53 ms to 324 ms).

Incorporating pitching motion into the kinematics has a major impact on the force dynamics. In Fig 20(A), while the overall trend of *α*_effective_ is similar to that for the flapping and stroke plane deviation combination, there are significant differences in the magnitude of the angles during the upstrokes. When the pitching motion is included, *α*_effective_ for the mid-inner and mid-outer wing chord lines on both wings range from -50° to ≈35°, whereas without the pitching motion, it varies from -125° to 25° on the outer wings and -100° to 20° on the inner wings. In addition to the differences in magnitude, which are prominent during the upstroke, the monotonic decreasing trend of *α*_effective_ during the downstroke is mitigated when the pitching motion is included: not only does the downstroke start with a high *α*_effective_ that decreases into the downstroke, but with pitching, *α*_effective_ subsequently increases to finish the downstroke as the inner and outer wing-sections start to supinate. One plausible explanation for the temporal variation of *α*_effective_ could be that the initial higher *α*_effective_ establish the LEVs early on during the downstroke, followed by a period of sustenance that necessitates lower *α*_effective_. Clearly, the pitching motion along the wing span (twisting of the wing) is used by the bat to maintain a nearly steady *α*_effective_ during the downstroke. Compared to the case without pitching, there is a more pronounced non-uniformity in *α*_effective_ between different sections of the left and right wings during the downstroke too, which is indicative of the wing pliability and ability to control force dynamics. During the 1^st^ upstroke, both wings exhibit a mostly uniform distribution of *α*_effective_ across the span, though there is still a weak inflection point apparent on the right outer wing. This is still evident on the right outer wing during the 2^nd^ upstroke, in addition to a stronger inflection point on the right inner wing at ≈225 ms.

The evolution of forces when pitching is added also differs significantly from the earlier settings. In Fig 20(B), towards the end of the 1^st^ downstroke, the higher pitching angle here amplifies the projected planform area, and increases lift further (and the drag). This feature is apparent during the 2^nd^ and 3^rd^ downstrokes as well, where a more sustained higher lift coefficient is evident, in addition to a negative net thrust. The high lift, which occurs near the beginning of the 2^nd^ and 3rd downstrokes for the cases without pitching, is sustained longer into the downstroke when pitching is included. This is attributed to better control of LEVs that form during each downstroke, and the pitch-up motion during supination at the end of each downstroke.

During the 1^st^ upstroke, the magnitude of the negative lift is reduced by nearly 50%, while a significant net thrust is apparent. A similar reduction in peak negative lift is also seen during the 2^nd^ upstroke, whilst an undulating variation in net thrust occurs during this time period. The most remarkable aspect of this upstroke is the large net thrust that is generated at ≈220 ms, which is attributed to the significantly higher *α*_effective_ on the inner right wing. The trend in the variation of lift and drag (or negative net thrust) are identical and opposite during the upstroke.

**Fig 21:**
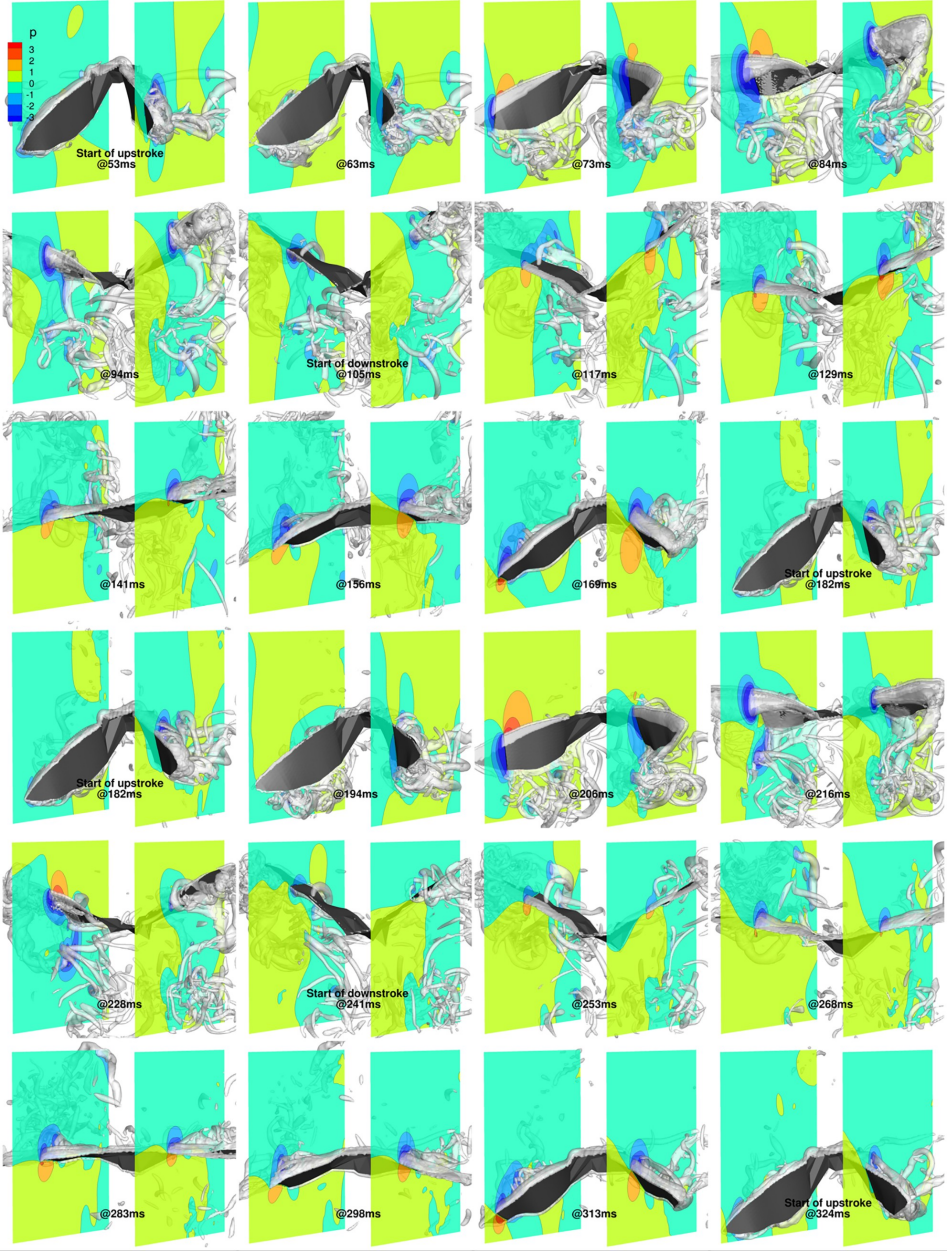
Combined flapping, stroke plane deviation and pitching (53 ms to 324 ms): 3D view of wing with pressure contours at y/c = 1.75 and y/c = -2, and isosurfaces of coherent vorticity (***ω***_***c***_ = 5).

Fig 21 shows the wing surface, coherent vorticity isosurfaces and pressure contours on approximate mid-span cutting planes, similar to Fig 17 and Fig 19, for the two flapping cycles from 53 ms to 324 ms. At the beginning of the upstroke, at 53 ms, the wing has a much larger pitch angle compared to the flapping motion case with stroke plane deviation (15° vs -50°). As a result, for a short amount of time (53 ms to ≈58 ms), the LEVs from the previous downstroke remain attached to the dorsal side of the wings. At 63 ms, the outer wings are pivoted almost vertically, and new LEVs form on the ventral side of the wings. From 63 ms to 73 ms, these LEVs grow stronger. From 73 ms to 84 ms, the wings undergo advanced rotation as they start to pitch down and translate upward to complete the upstroke. From 84 ms to 94 ms, the LEVs reduce in strength and are stabilized, during which the entire left and right wings are surrounded by relatively low pressure bubbles. Beyond 94 ms, up until 105 ms, the wings continue pitching down while flapping up, and in doing so, the LEVs weaken further and glide under the ventral side of the wings to form low pressure bubbles for the downstroke.

At the start of downstroke, at 105 ms, the wings have completed their pronation and their ventral sides are exposed to the freestream at a positive *α*_effective_. From 117 ms to 156 ms, the entire wing section pitches down, though non-uniformly. Both outer wings and the left inner wing change *ψ* gradually until about 169 ms, whereas the right inner wing exhibits a rapid decrease until 129 ms, followed by a gradual increase in *ψ* until 169 ms. During this period, the LEVs not only stay attached to the dorsal side of the wings, but they grow stronger. Beyond 169 ms, the rapid pitch-up motion on both wings cause the LEVs to slide off and separate out from the dorsal side of the wings. From 169 ms to 182 ms, towards the end of the downstroke, the wings perform a fast supination to transition to the next upstroke.

During the 2^nd^ upstroke, from 182 ms up until 206 ms, there is a similar trend in the variation of *α*_effective_ as the 1^st^ upstroke, where LEVs form on the ventral side and grow in strength. Over the next 10 ms, there is a vastly different trend in *α*_effective_ that translates to different pressure signatures over the wings: whereas the left inner and outer wings exhibit a more consistent change in *α*_effective_, the angle at the inner right wing suddenly increases at a faster rate than the outer wing. This results in a reduced projected area of the right wing, which mitigates the peak negative lift at 216 ms. At about this time, the inner right wing undergoes a sudden pitch up motion reducing *α*_effective_ from a high negative value to a positive value (this is not evident at 84 ms). As a consequence, the stagnation high pressure region forming near the dorsal trailing edge of the right wing at 84 ms does not materialize at 216 ms, thereby flipping the negative lift to a positive lift. Instead, a high pressure region materializes at 228 ms (but not at 94 ms), causing a sudden spike in net thrust. At around this time (still at 228 ms), the original LEVs also separate from the ventral side of the wings followed by the formation of weaker LEVs, which reinstate the negative lift and positive net thrust. This extent of unsteadiness is apparent only in the 2^nd^ upstroke where the sudden pitch-up movement occurs at ≈220 ms. Towards the end of the upstroke, the wings rapidly pitch down to get ready for the downstroke. The downstroke from 241 ms to 324 ms is nearly identical to the previous downstroke from 105 ms to 182 ms, and the inner right wing pitches in-phase with the rest of the wing, leading to a more steady distribution of positive lift.

#### Combined flapping, stroke plane deviation, twisting and cambering

**Fig 22:**
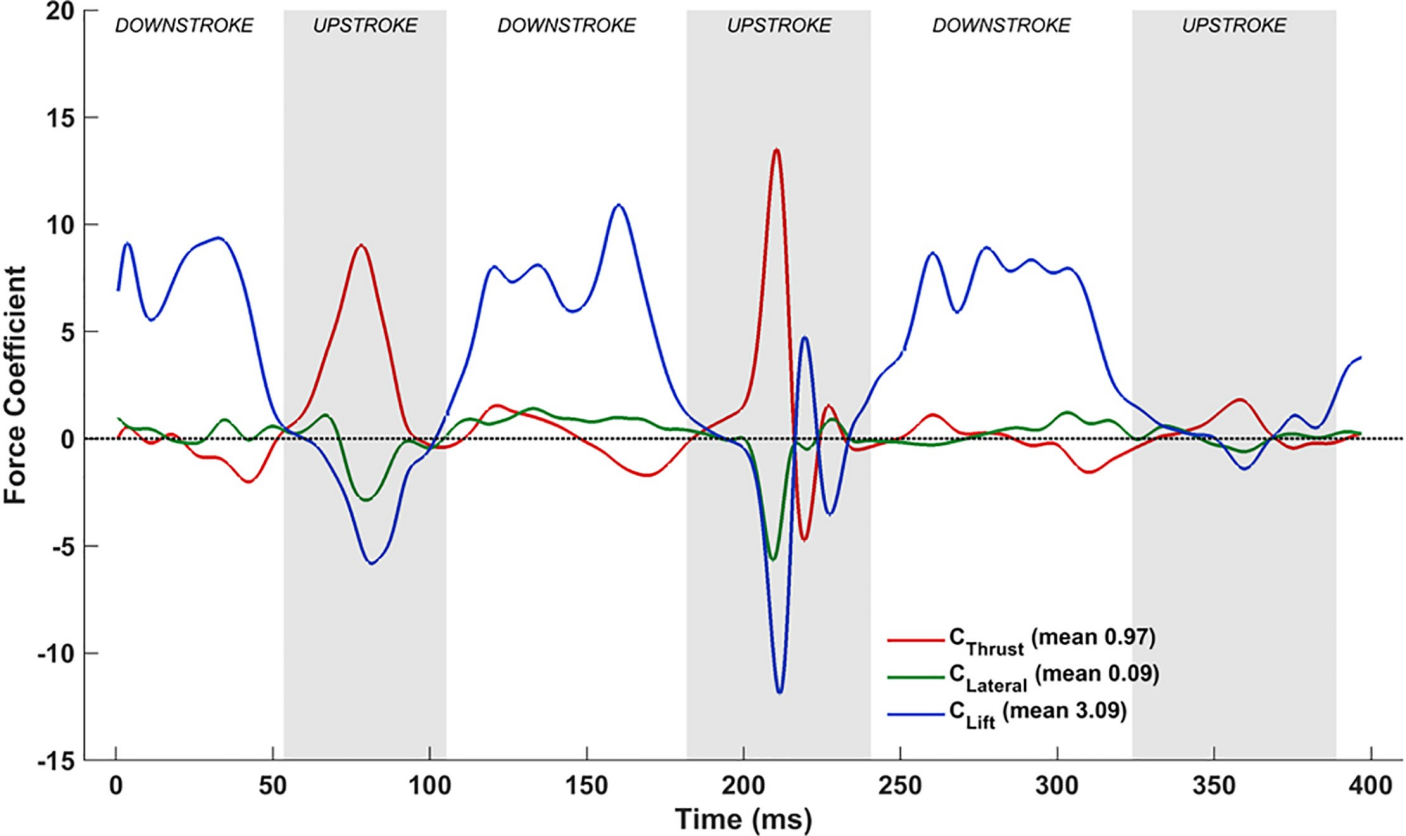
Combined flapping, stroke plane deviation, pitching and cambering: Forces evolution averaged over two flapping cycles (53 ms to 324 ms).

By definition, the inclusion of chordwise camber (**C**_i,j,n_) does not change the temporal evolution of *α*_effective_, which remains the same as in Fig 20(A). However, similar to classical airfoil theory, the introduction of chordwise camber does impact force production, as seen by comparing Fig 22 and Fig 20(B). Although the trends in force production exhibited by the introduction of camber are very similar without it, there are subtle differences in the magnitudes. During the 1^st^ upstroke (53 ms to 105 ms), when chordwise cambering is included, both negative lift as well as the peak net thrust are reduced. A similar trend is evident for the upstroke between 182 ms and 241 ms.

**Fig 23:**
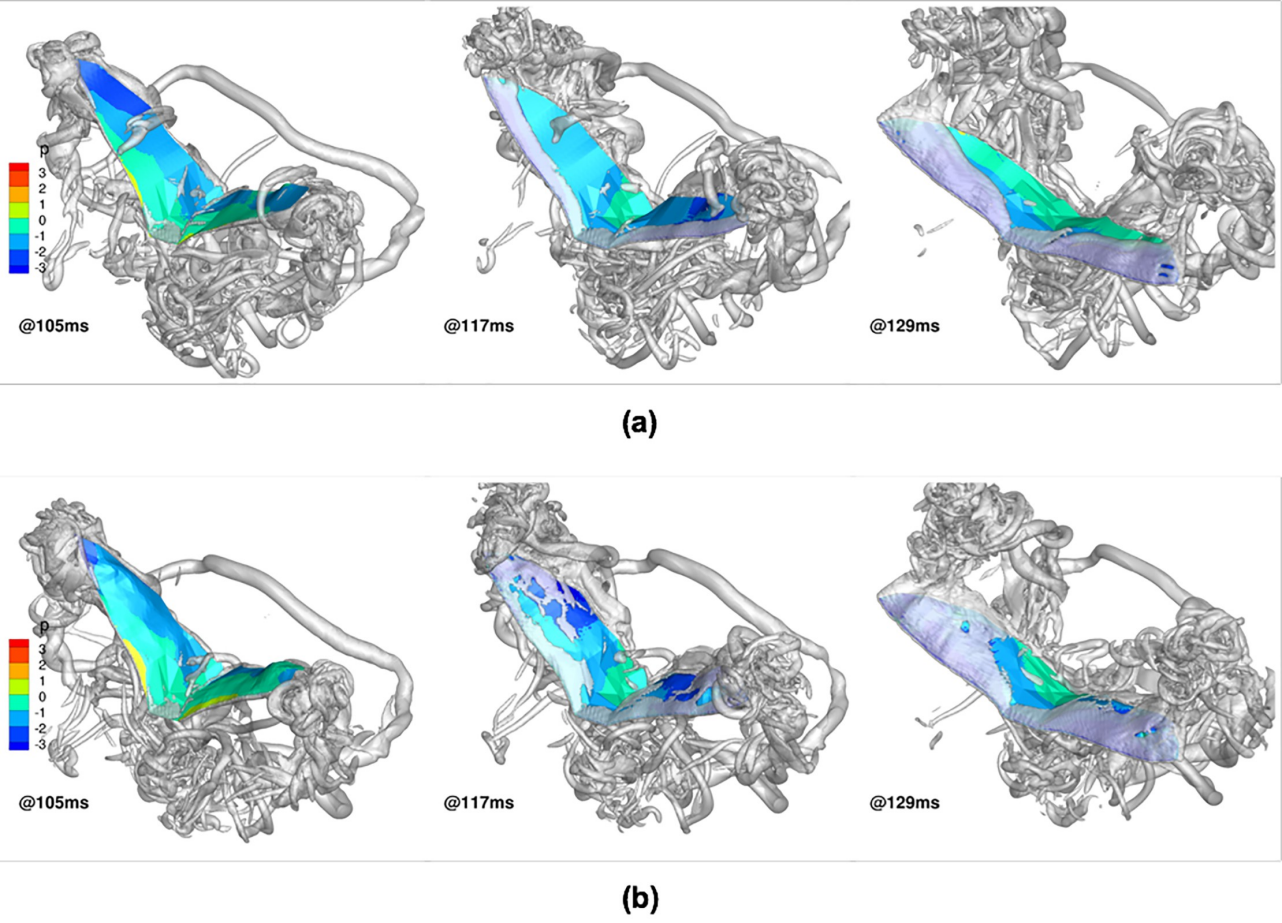
Effect of cambering on evolution of dorsal LEV during downstroke: wing surface colored by pressure, isosurfaces of coherent vorticity (*ω*_*c*_ = 5): (a) without camber, (b) with camber.

During the downstroke, the trends are similar to that without camber, but there is an increase in the lift force: the chordwise camber forces the LEVs to stay in close proximity of the dorsal surface to cover the majority of the bat wing, while the LEVs on wings without cambering separate more easily. Fig 23 shows this phenomenon during the downstroke between 105 ms and 129 ms. It is evident that the LEVs when **C**_i,j,n_ is included spread out more over the dorsal surface, resulting in lower pressure over a larger area of the wing (at 129 ms). The attached LEVs during the downstroke, together with the reduction in peak negative lift during upstroke, boost the average lift force by nearly 35% with the inclusion of camber. This was also observed by Gopalakrishnan [62] in his investigation of a flexible wing with aerodynamic force induced chordwise camber. It is noteworthy that camber decreases the net thrust by 20% for the same reasons that the negative lift decreases during the upstroke.

#### Combined flapping, stroke plane deviation, twisting, cambering and flexion

**Fig 24:**
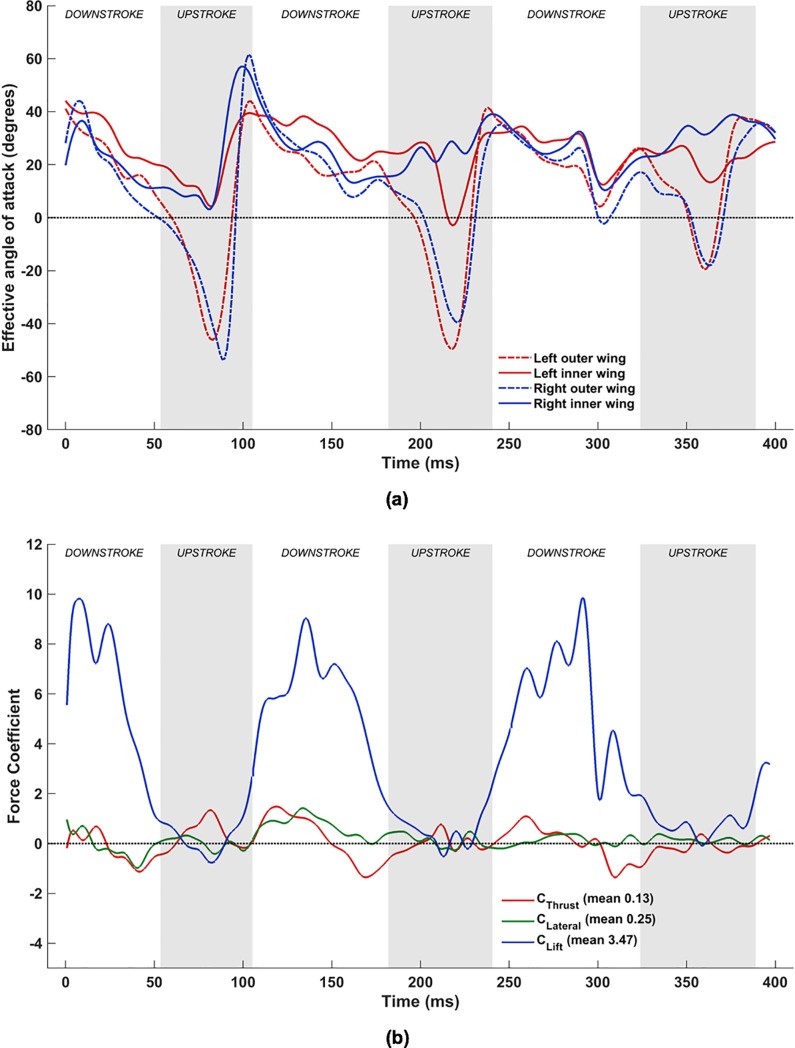
Combined flapping, stroke plane deviation, pitching, cambering and flexion: (a) time variation of *α*_effective_, (b) Forces evolution averaged over two flapping cycles (53 ms to 324 ms).

With the inclusion of flexion (**D**_i,n_), the complex wing kinematics of the *H*. *pratti* is represented in its entirety. Fig 24(A) shows the time evolution of *α*_effective_ at the previously defined four spanwise locations on the wings, and Fig 24(B) shows the corresponding force evolution in time. When comparing Fig 24(A) with the set-up without flexion (Fig 20(A)), a notable difference is the nearly universal positive *α*_effective_ at the inner wing during the upstroke when flexion is included.

**Fig 25:**
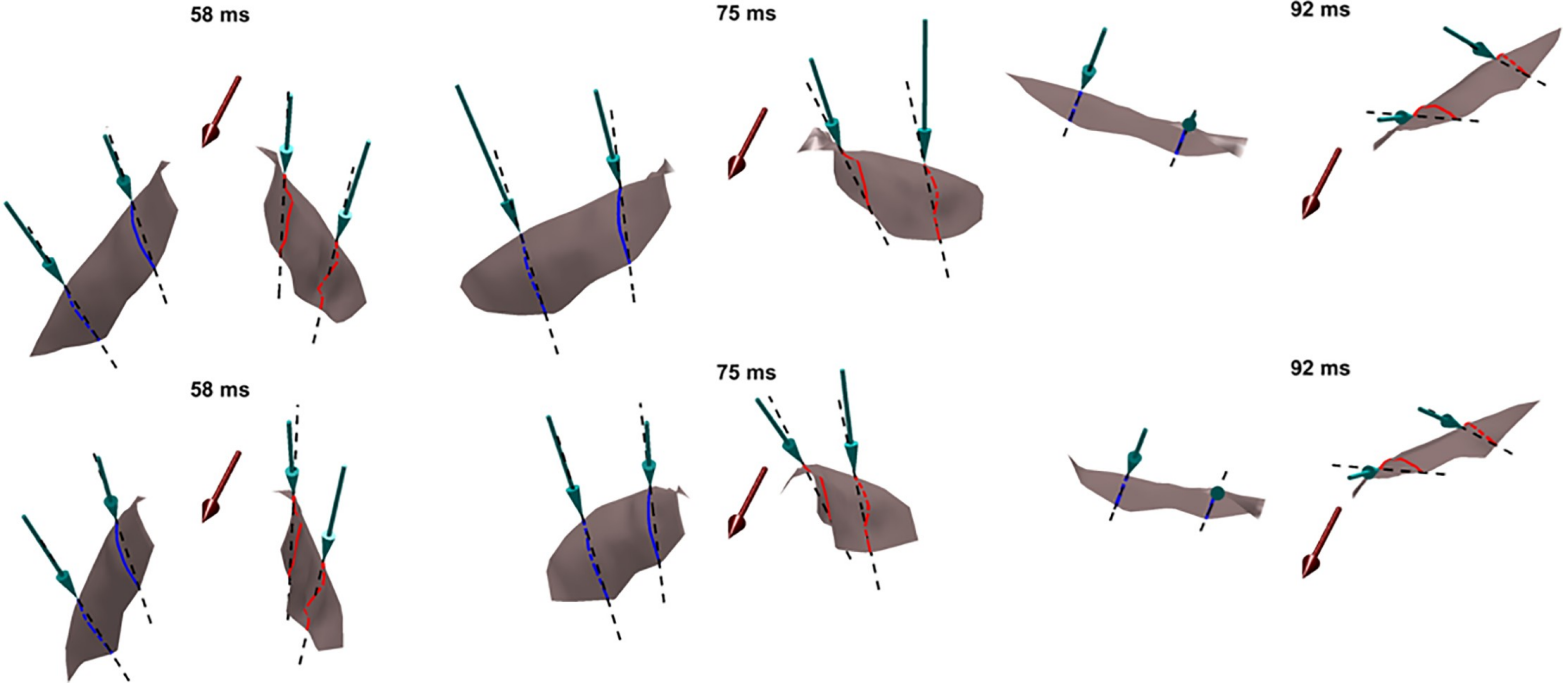
Effective angle of attack, *α*_effective_, at mid-inner and mid-outer wing locations (top–without flexion, bottom–with flexion), with maroon arrows denoting flight direction, green arrows denoting the direction of the local relative flow velocity at the leading edge of each airfoil, and dashed lines denoting extensions of chord lines at each airfoil section.

To explain this in more detail, Fig 25 tracks the evolution of the wing surface and the orientations of the mid-inner and outer wing sections on both wings during the upstroke of the 1^st^ flapping cycle. These are compared with snapshots at the same time instants without flexion. From 58 ms until 92 ms, both wings are brought inwards towards the body of the bat and flapped upwards. When flexion is not included, during the early stage of the upstroke at 58 ms, *α*_effective_ is ≈-30° for all wing sections (see Fig 20(A)). When flexion is incorporated, at the inner wing sections, ${\vec{u}}_{effective}$ flips over to the opposite side of the chord line and changes sign, with a corresponding *α*_effective_ of about +15°. This difference is more pronounced at 75 ms, when the wings are aligned to the direction of the upstroke, and slice through almost vertically upward in the stroke plane and backward. Whereas without flexion, *α*_effective_ is ≈-50° for all four airfoil sections, with flexion *α*_effective_ is ≈+5° at the inner wing sections. Towards the end of the upstroke, at 92 ms, the differences are marginal, and barely discernible in Fig 25. Thus, during the upstroke, flexion not only reduces the planform area of the wing but also works to achieve a positive angle of attack at the inner wing section. By orientating the inner wing such that *α*_effective_ is positive on most of the wing, negative lift (and positive net thrust) are eliminated as evidenced in Fig 24(B).

An additional feature that stands out is the sharp drop in *α*_effective_ at the left mid- inner wing section at approximately 205 ms from a positive value of about 25° to a small negative value in a relatively short time span of ≈15 ms, followed by a rapid recovery back to 30° in the next ≈15 ms (Fig 24(B)). Interestingly, this coincides with about a 5° to 10° excursion in *α*_effective_ of the right inner wing section in the opposite direction. The consequence of these variations in *α*_effective_ on the left and right wings is that the oscillatory behavior of lift and net thrust in the middle of the upstroke (present in Fig 20(B) and Fig 22 at ≈220 ms) during the 2^nd^ flapping cycle is eliminated. Thus, the oscillatory behavior of the forces at ≈220 ms without flexion is an artifact only because flexion was neglected in the kinematics. This aligns with the hypothesis that the instantaneous rate of flapping, pitching, and flexion work in tandem to effect a near zero negative lift penalty during the upstroke.

**Fig 26:**
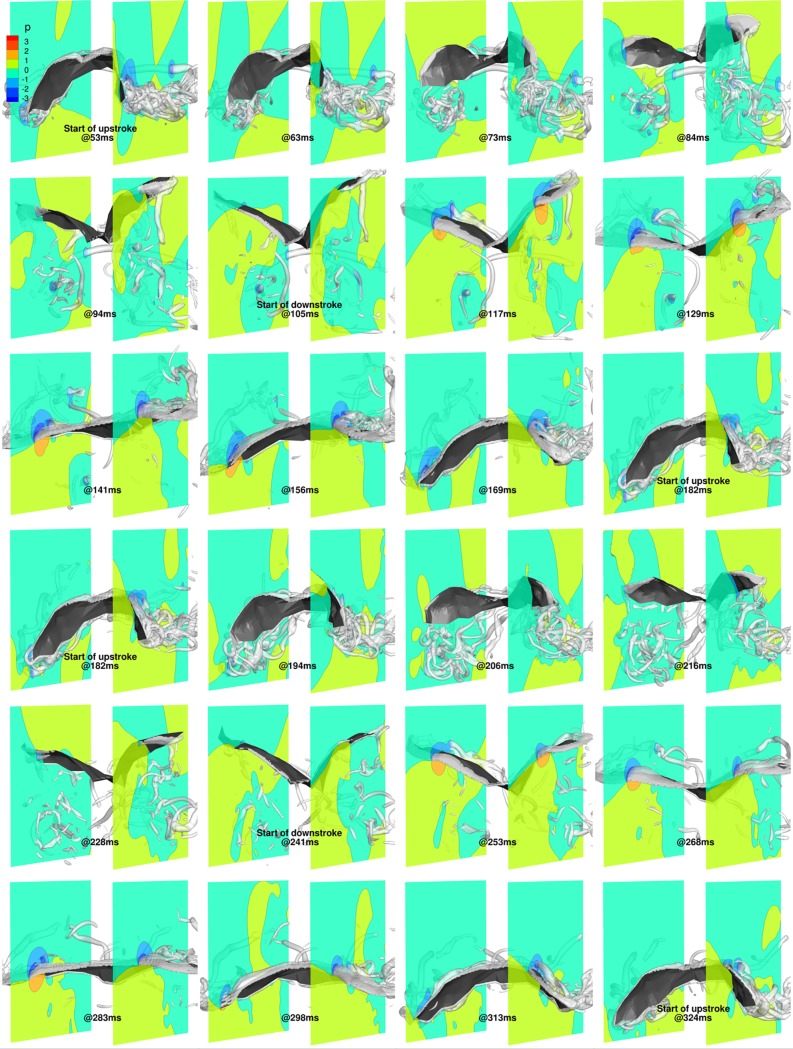
Combined flapping, stroke plane deviation, pitching, cambering and flexion (53 ms to 324 ms): 3D view of wing with pressure contours at y/c = 1.75 and y/c = -2, and isosurfaces of coherent vorticity (***ω***_***c***_ = 5).

Fig 26 shows the vorticity and pressure contours from 53 ms to 324 ms for the combined kinematics that includes *ϕ*,*θ*,*ψ*, **C**_i,j,n_ and **D**_i,n_. For this case, at 53 ms and 184 ms, the wings start the upstroke with the tips drawn-in towards the body. From 53 ms to 84 ms (and 182 ms to 216 ms during the 2^nd^ flapping cycle), the inner part of the wing reaches a nearly vertical orientation due to flexion. During this phase of motion, the outer part of the wing continues to benefit from the pitching motion, and maintains a nearly vertical profile for the initial part of the upstroke (53 ms to 73 ms and 182 ms to 206 ms during the 1^st^ and 2^nd^ upstroke, respectively, as explained previously when pitching is incorporated into the kinematics). Essentially, both pitching and flexion work in tandem to reduce the planform area and produce positive *α*_effective_ during the upstroke to minimize the peak negative lift by an order of magnitude of the value when both these movements are excluded (peak *C*_Lift_ ≈ -1 vs -10 during the 1^st^ flapping cycle).

One of the distinguishing features of Fig 26 is the complete lack of extreme pressure fluctuations surrounding the wing during the upstrokes. During the upstrokes, although LEVs form on the ventral side of both outer wings, their lifespan is much shorter and strength much weaker than the LEVs that are formed without flexion. When combined with wing twist, the combined kinematics result in largely muted force fluctuations. Between the 1^st^ and 2^nd^ upstroke, the ventral LEVs on the outer wings are even weaker during the 2^nd^ flapping cycle. The low-strength LEVs dissipate during the end of the upstroke.

Flexion also affects force production during the downstroke, but not as drastically as the upstroke. The multiple peaks in the lift forces from 105 ms to 169 ms and 241 ms to 313 ms (Fig 24(B)) result from the combination of multiple LEVs that form and shed during the downstrokes, the presence of tip vortices during the second half of the donwnstrokes and wing orientation, as discussed in some detail in Windes et al.[52].

## Summary and conclusions

This paper investigates the nearly straight and level flight of an insectivorous bat (*H*. *pratti*). The relationship between flight kinematics and force dynamics is established through the decomposition of the complex 3D wing motion into basic descriptors of flapping flight. The novel framework reduces the kinematics into physical movements that include flapping, stroke plane deviation and pitching, together with cambering and flexion. To further elaborate on the aerodynamics, the effective angle of attack is revisited, and used as the basis for interpreting the unsteady flow physics. While it is well known that bat wings are highly articulated and the kinematics, therefore, complex, this is the first attempt to break down the intricate motion into simpler physically relatable components of flapping flight. Both the left and right wings are initially considered rigid, and the first two movements, flapping (*ϕ*) and stroke plane deviation (*θ*), describe canonical flapping flight. The third movement (*ψ*) represents the twisting motion of the wing. Here, both wings are flexible, and are modeled as a collection of chord line sections incident at different pitching angles. This representation is further extended to account for cambering at each chord line. Finally, flexion models the displacement of each of these airfoils within the enclosing plane, and out of each plane. The decomposition is combined with aerodynamic simulations to investigate the cumulative effect of these movements on force production, and their primary contribution to the unsteady vortex dynamics.

For the nearly straight and level flight investigated, the stroke plane angle was calculated to be 53°, with a stroke amplitude of around 90°. The wing motion exhibited approximately ±9° and ±13° mean deviation from the stroke plane for the right and left wing, respectively, with the left wingtip subtending a clockwise crescent (looking outward from the body). The pitch angle varied substantially, both temporally and spatially along the span of the wing in the form of twist. During the downstroke phase, it decreased from varying between 50° and 75° at the root to varying between 30° and 50° at the tip. Following the advanced rotation at the end of the downstroke, an opposite twisting trend was evident during the upstroke, when the pitch angle increased from varying between 80° and 110° at the root to varying between 125° and 130° at the tip. Cambering of airfoil sections along the wing span reached a maximum of 30–40% of the standard mean chord, while flexion varied between 10-50mm during the flapping cycles, with an average of approximately 35mm, and reached 50mm during the middle of the upstroke.

Aerodynamic simulations were performed using the IBM with a background mesh of 32 million cells and a wing surface defined by approximately 70,000 surface elements. By studying the cumulative aerodynamic effects of different physical movements, the following findings were substantiated for the nominally straight and level flight of *H*. *pratti*:

1. The flapping motion by itself was aerodynamically ineffective because of the large negative lift produced during the upstroke. Overall, this motion recovered only 17% of the mean lift generated by the native kinematics and produced a net drag force, which indicated that the flapping motion by itself is not very effective, and requires other components of kinematics for effective flying;
2. Stroke plane deviation had a relatively small impact on lift when merged with flapping (combination accounted for 25% of the mean overall lift), but this motion managed to nullify the drag force generated by the flapping motion;
3. The addition of pitching and wing twist reduced negative lift during upstroke, and redirected some of the pressure differential to net thrust production. Supination allowed wings to position themselves such that the force components generated by the LEVs in the vertical direction were inconsequential, while at the same time produced a large component of net thrust. Another benefit of the pitching motion was the reduction of *α*_effective_ during the downstroke, which sustained the growth of LEVs on the dorsal side of the wing for longer periods of time. This motion combination accounted for 66% of the mean overall lift, and an effective mean net thrust that was nearly an order of magnitude higher than the overall value;
4. Cambering stabilized LEVs during the downstroke and allowed them to glide over the dorsal side of the wings, delaying separation from the surface. This combination accounted for nearly 90% of the mean overall lift;
5. Flexion allowed the bat to fine-tune *α*_effective_ during the upstroke such that the negative lift produced at the outer wing was nullified by the positive lift produced at the inner wing, resulting in a near zero lift during the upstroke;
6. From this study, it is notable that the aerodynamic effective angle of attack, *α*_effective_, emerged as the most significant parameter that affected the instantaneous lift and net thrust production.

Conclusions made regarding the contributions of each movement to force production are somewhat dependent on the order in which each motion is incorporated into the kinematics, and on the flight regime. The sequence followed in this paper is that of increasing complexity, from plain flapping motion to the complete complex kinematics. Many of the physical movements seem to work in synchrony, although each one individually may not have a favorable impact on the aerodynamics. However, such experimentation, which is made possible by the proposed decomposition, could result in novel combinations of movements for various flight regimes. The reduction to these foundational parameters from complex flight could facilitate comparison not only to the vast body of literature featuring canonical flight experiments and computations (flapping of rigid and flexible plates, pitching and/or plunging airfoils, etc.), but also comparison of wing kinematics and aerodynamics across different flying animals and flight regimes.

## Supporting information

S1 Text Outline for the decomposition of native kinematics. Information regarding motion capture of the flight kinematics is presented in Windes et al. [52]. The following is a step-by-step procedure to decompose the recorded native kinematics into different physical movements already described. This involves two main steps: first, the stroke plane location and orientation are determined based on the time-series of the wingtip points for both the left and right wings and the time-series of the shoulder points. This is followed up the decomposition process, which takes the instantaneous location information of the bat wings to isolate various components of motion:
Determining the stroke plane:
Locate the time-series of should point positions, and determine the origin (*O*) of the body fixed coordinate system (**x**_**b**_, **y**_**b**_, **z**_**b**_)Locate the time-varying wingtip positions of both wings, and project the points onto the vertical bisecting planePerform a linear regression on these points, and determine the stroke plane angle, *β*, and the stroke plane (**x**_**b**_, **y**_**b**_)Decomposition of the native kinematics:
Determine instantaneous span lines as the lines joining *O* and the wingtipsDivide both span lines into a predetermined number of planes (**x**_**a**_, **z**_**a**_) that are perpendicular to the span linesDetermine airfoil sections as the intercept of the wing surface and the previously identified planes
The line joining the leading edge and trailing edge of each airfoil is the chord lineFlexion is determined as the offset of the quarter-chord from the span lineCamber is determined as the offset of the airfoil from the chord lineThe local pitching angle is determined as the angle between the chord line and **x**_**a**_ in the airfoil plane (**x**_**a**_, **z**_**a**_)Stroke plane deviation is the angle between the span line and the stroke planeThe flapping angle is the angle between the span line and **y**_**b**_ in the stroke plane (**x**_**b**_, **y**_**b**_).
(TIF)

S1 Video Straight and level flight of Pratt’s roundleaf bat (Re = 400): Flapping motion. (MP4)

S2 Video Straight and level flight of Pratt’s roundleaf bat (Re = 400): Flapping motion with stroke plane deviation. (MP4)

S3 Video Straight and level flight of Pratt’s roundleaf bat (Re = 400): Flapping and pitching with stroke plane deviation. (MP4)

S4 Video Straight and level flight of Pratt’s roundleaf bat (Re = 400): Flapping and pitching with stroke plane deviation and cambering. (MP4)

S5 Video Straight and level flight of Pratt’s roundleaf bat (Re = 400): Flapping and pitching with stroke plane deviation, cambering and flexion (native kinematics). (MP4)

S6 Video Straight and level flight of Pratt’s roundleaf bat (Re = 1200): Flapping and pitching with stroke plane deviation, cambering and flexion (native kinematics). (MP4)

S7 Video Straight and level flight of Pratt’s roundleaf bat (Re = 12000): Flapping and pitching with stroke plane deviation, cambering and flexion (native kinematics). (MP4)

S8 Video Animations of perspective view of background mesh (plotting every 5th grid line) for fluid simulation enclosing the bat and overlaid with isosurfaces of coherent vorticity (), with orthographic projections of top, front and side views (Re = 400). (MP4)
