# Supplementary figures and images for: Canonical description of wing kinematics and dynamics for a straight flying insectivorous bat (Hipposideros pratti)
Source: PLoS One. 2019 Jun 25;14(6):e0218672. doi: 10.1371/journal.pone.0218672 (PMC6592571; doi:10.1371/journal.pone.0218672)

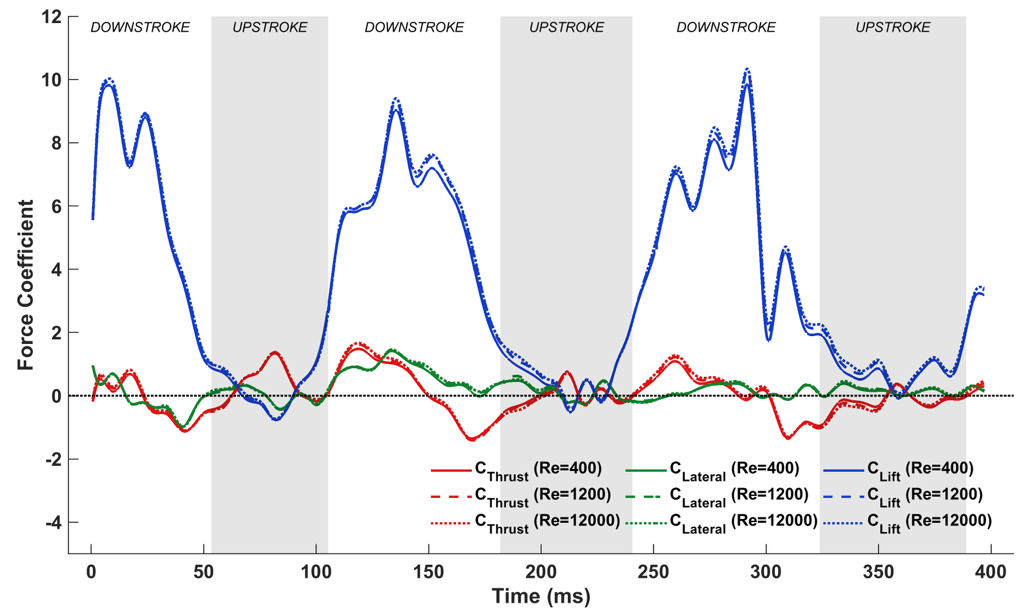

Supplement: S1 Text — Information regarding motion capture of the flight kinematics is presented in Windes et al. [52]. The following is a step-by-step procedure to decompose the recorded native kinematics into different physical movements already described. This involves two main steps: first, the stroke plane location and orientation are determined based on the time-series of the wingtip points for both the left and right wings and the time-series of the shoulder points. This is followed up the decomposition process, which takes the instantaneous location information of the bat wings to isolate various components of motion: Determining the stroke plane: Locate the time-series of should point positions, and determine the origin (O) of the body fixed coordinate system (xb, yb, zb)Locate the time-varying wingtip positions of both wings, and project the points onto the vertical bisecting planePerform a linear regression on these points, and determine the stroke plane angle, β, and the stroke plane (xb, yb)Decomposition of the native kinematics: Determine instantaneous span lines as the lines joining O and the wingtipsDivide both span lines into a predetermined number of planes (xa, za) that are perpendicular to the span linesDetermine airfoil sections as the intercept of the wing surface and the previously identified planes The line joining the leading edge and trailing edge of each airfoil is the chord lineFlexion is determined as the offset of the quarter-chord from the span lineCamber is determined as the offset of the airfoil from the chord lineThe local pitching angle is determined as the angle between the chord line and xa in the airfoil plane (xa, za)Stroke plane deviation is the angle between the span line and the stroke planeThe flapping angle is the angle between the span line and yb in the stroke plane (xb, yb). (TIF) [file pone.0218672.s001.tif]
